# Deployment-related quarantining—a risk or resilience factor for German military service members? A prospective analysis during the third–fifth waves of COVID-19

**DOI:** 10.3389/fpubh.2023.1267581

**Published:** 2023-12-13

**Authors:** Antje H. Bühler, Gerd-Dieter Willmund

**Affiliations:** Bundeswehr Center for Military Mental Health, Military Hospital Berlin, Berlin, Germany

**Keywords:** quarantine, military deployment, risk and resilience factors, mental health, military culture

## Abstract

**Background:**

Mandatory deployment-related quarantining added further constraints on soldiers during the pandemic. Contrary to overwhelming research documenting an adverse impact of quarantining on mental health, no adverse short-term mental health effects of pre-deployment quarantining for German soldiers were identified. Therefore, we are interested in a potentially delayed onset, the impact of an additional post-deployment quarantine, and quarantine-associated risk and resilience factors predicting mental health post-deployment.

**Methods:**

In a prospective research design, 928 German soldiers enrolled in the study at the in-processing of pre-deployment quarantine between February 2021 and March 2022. Every German military service member undergoing pre-deployment quarantine could participate. The soldiers were between 18 and 64 years old; 87.5% identified as male and 12.5% as female. Self-reported mental health (Mini-SCL), perceived social support (FSozU-K22), and perceived unit cohesion were assessed three to five times: at the beginning and the end of pre-deployment quarantine (*N*_t1_ = 928, *N*_t2_ = 907), if still mandatory—at the beginning and the end of post-deployment quarantine (*N*_t3_ = 143 and *N*_t4_ = 132), and 3 months post-deployment, on average 7 to 8 months later than pre-deployment quarantine (*N*_t5_ = 308). The analyzed quarantine-associated risk and resilience factors were informedness about COVID-19, infection risk, quarantine benefit, clarity of quarantine protocol, need for intimacy/bonding, norms, stigma, practicality, financial disadvantages, boredom, and health-promoting leadership.

**Results:**

Despite four different mental health trajectories identified, repeated measures ANOVAs revealed a significant improvement in mental health post-deployment (*F*[2,265] = 21.54, *p* < 0.001), a small decrease in social support (*F*[2,266] = 16.85, *p* < 0.001), and no significant changes in unit cohesion (*F*[2,264] = 0.482, *p* = 0.618) 3 months post-deployment. Using stepwise regression, 24% of variance in mental health symptomatology post-deployment is predicted pre-deployment by a clear quarantine protocol, unit cohesion, intimacy/bonding, and social support (*F*[4,263] = 22.23, *p* < 0.001). In total, 30% of mental health at the end of post-deployment quarantine is predicted by stigma and a clear quarantine protocol (*F*[2,99] = 22.22, *p* < 0.001).

**Conclusion:**

Although no overall adverse impact of quarantining on mental health was found, it is recommended to address perceived stigma and clearly communicate the quarantine protocol, and to further follow up on the perceived decrease in social support.

## 1 Introduction: German armed forces and the pandemic

Requests for military support by overburdened civilian administrations in coping with the health crisis caused by the COVID-19 pandemic have shaped the armed forces in unprecedented ways. This relates to both the fulfillment of new mandates and how primary military tasks had to be adapted to pandemic requirements.

From 19 March 2020 to 31 March 2022, 111,000 personnel of the German Armed Forces (GAF) were deployed to support the German healthcare system with a maximum of 19,000 personnel simultaneously employed on 15 February 2021 (1). This happened amidst the strength of the German Armed Forces of 182,140 personnel and the main task of the military at the time—keeping up deployment commitments worldwide, with contingents of about 4,500 military service members in 2021, requiring regular troop rotations.

However, troop rotations during the First World War are posited to have been one of the main driving factors for the pandemic of the Spanish flu (2) and have even been proven to be the reason for the outbreak of the cholera epidemic in Haiti (3–5). Thereby, rotations posed a health threat to vulnerable populations that lacked immunization status and healthcare during the COVID-19 pandemic. At the same time, the same troop rotations were necessary to avoid a power vacuum in war-torn countries, heightening the risk of further destabilization and human rights violations. Health concerns had to be reconciled with security concerns for the vulnerable populations in conflict-ridden countries. Throughout the world, Departments/Ministries of Defense addressed this dilemma by ordering the implementation of different containment measures for deploying military service members during the COVID-19 pandemic (6–8) in line with World Health Organization (WHO) guidelines (9–11). One of the main non-pharmacological hygiene measures for rotating troops was quarantining: Before being deployed, personnel were quarantined individually in separate hotel rooms for 2 weeks (pre-deployment quarantine). Upon returning home, they underwent home-based quarantining for another 2 weeks (post-deployment quarantine) (7, 8, 10). This imposed deployment-related quarantining has prompted complaints by affected soldiers to the Parliamentary Commissioner of the Bundeswehr (12). In addition, meta-analyses and reviews reach the common conclusion that quarantining and isolation have an adverse mental health impact (13–16). This starting point has sparked our interest in (a) how deployment-related quarantining has impacted mental health in addition to military deployment and (b) how a potential impact could be addressed.

### 1.1 Military mental health: military deployments and the pandemic

Soldiers are considered an especially resilient occupational sub-population. They undergo regular thorough medical examinations of their general fitness to serve, including deployment-driven medical assessment (17, 18). At the same time, it is a population subgroup that has to endure duty-specific constraints and infringements, such as regular repostings and long commutes, separation from family and friends due to training and deployments, and deployment-related stressors and traumatic events, mainly in combat.

Different prevalence and incidence rates for mental disorders of military personnel have been identified depending on posting country, region of deployment, and methodology. For instance, the prevalence of PTSD is higher for Iraq (12.9%; 95% CI 11.3 to 14.4%) than for Afghanistan (7.1%; 95% CI 4.6 to 9.6%) for UK and US military personnel (19). Prevalence rates for mental health disorders among German military personnel are not higher than civilians in general (20). Growing research, also for German soldiers, indicates that it is not deployment *per se* but combat with a heightened threat to life and physical integrity that is linked to elevated prevalence rates for anxiety disorders and PTSD (19, 20). As for differential trajectories of mental health across the deployment cycle, three trajectories were identified, namely, a resilient-stable trajectory (83.8%), an increase in mental distress (9%), and a decrease in mental health symptoms (7.1%), so far only analyzed for US military personnel (21).

Few studies have analyzed the impact of the pandemic on military mental health: Considerable resilience and adaptation to the pandemic have been found for activated National Guard (NG) personnel in the US (22), medical staff of German military hospitals (23), and the majority of 3,078 US military veterans, but 13.2% of US veterans reported a clinically meaningful increase in distress in a prospective cohort survey (24). By comparison, in civilian research on reactions to macro-stressors, also for the pandemic, one additional trajectory has been identified: chronic-stable (25, 26).

As for *specific military resilience* factors during the COVID-19 pandemic, the few existent studies only based on US military service personnel showed that perceived unit cohesion, supportive leadership, and health-promoting leadership lessened the risk for reporting PTSD and anger, and clinically significant anxiety and depression (22, 24, 27), regardless of the personnel’s active response to the pandemic (22). Coping style and regulatory focus were identified as individual resilience factors for Chinese military officers during the COVID-19 pandemic (28). Identified *risk factors* for UK and US military veterans during the pandemic were higher levels of PTSD (29), a pre-existing mental health disorder (30), and medium or heavy combat exposure (29).

### 1.2 Impact of quarantining on psychosocial wellbeing

So far, there is a dearth of research on the mental health impact of deployment-related quarantining, while there has been substantial research on the impact of civilian quarantining and isolation measures (13–16). Adverse mental health effects of quarantining and isolation were found across all meta-analyses and systematic and rapid reviews for civilian quarantining, including anxiety and depressive disorders and stress-related disorders (13–16); and sampled groups moderated the relationship between quarantining and mental health (16). None of them were based on prospective or longitudinal research designs. However, the comparison of meta-analyses and systematic reviews on home confinement and lockdowns provides evidence that those based exclusively on longitudinal and prospective research designs (31) find more heterogeneous outcomes and smaller effect sizes than those mostly including cross-sectional studies (32–34). Therefore, we conclude that it is paramount to adapt a prospective longitudinal research design.

The only two studies, to our knowledge, studying military mental health associated with deployment-related quarantining have not found indications for higher incidence or prevalence rates of mental disorders following quarantining (35, 36), although a considerable percentage of sleeping problems was reported (32%) in one study (35, 37), which is often a precursor for mental health disorders (38–41). At the same time, differing trajectories of more vulnerable groups might be masked, when only statistic differences in means are explored (25, 26). Identifying vulnerable groups early on is necessary for enabling in-time support to them.

Therefore, we are interested in the following questions:

Question 1: Is there a delayed onset of mental health symptomatology post-deployment in the long term?

Question 1a: Which resilience trajectories can be identified for military deployment shaped by quarantining?

Question 1b: Do they resemble the three resilience trajectories identified for deployed US soldiers (21) or the more common four trajectories as a response to the pandemic (26)?

Most of the studies focus on how the health protective factors facilitate mental health during the pandemic, including general perceived social support or its military form, unit cohesion (26, 42). There is a dearth of research on how perceived social support or unit cohesion as indicators of social wellbeing have been affected by physical isolation, such as in quarantining or confinement. In an analysis of changes in social wellbeing during pre-deployment quarantining, no short-term changes have been found, with the exception of small significant interaction effects (but no main effects) for age and rank: Perceived social support decreased more for younger soldiers, while perceived unit cohesion relatively increased for enlisted personnel (lowest rank), decreased relatively for non-commissioned officers, and remained relatively stable for officers (36). As decreased social wellbeing can also be a precursor for mental health problems, we are interested in its long-term development:

Question 2a: Does perceived social support change across the complete deployment cycle?

Question 2b: Does perceived unit cohesion change across the complete deployment cycle?

#### 1.2.1 Pre-deployment and post-deployment quarantining

Quarantining conditions differed substantially between pre- and post-deployment quarantine. Though during pre-deployment quarantine soldiers were isolated individually in single hotel rooms, the quarantining in one hotel was a collective shared experience, like deployment itself. The practicalities were fully cared for, including medical care and a psychological hotline.

At the time of post-deployment quarantine, all quarantinees had previous quarantining experience as they had at least undergone pre-deployment quarantining. Post-deployment at-home quarantine was similar to the experience of civilian quarantining. However, post-deployment quarantining neither affected monthly pay nor employment status nor was it related to being infected or having been in contact with an infected person. Therefore, we are interested in changes in psychosocial wellbeing across pre- and post-deployment quarantine:

Question 3: Does the level of mental health change across pre- and post-deployment quarantining?

Question 4: Does perceived social support in general change across pre- and post-deployment quarantining?

Question 5: Does perceived unit cohesion change across pre- and post-deployment quarantining?

Regardless of the impact of specific quarantining measures, we are interested in changes in psychosocial wellbeing over the course of the pandemic.

Question 6: Does psychosocial wellbeing change with the duration of the pandemic?

### 1.3 Quarantine-related risk and resilience factors

In our study, we are mainly interested in quarantine-specific risk and resilience factors, which can be addressed by the organizational health policy, military leadership, and non-mental health experts, mainly soldiers. As a consequence, we focus on perceived external factors of policy and leadership information, communication, and implementation characteristics of quarantining (for definitions of risk and resilience factors, please see the glossary in Supplementary material 1).

In substantive research (13–16), a number of quarantine-specific risk factors have been identified: previous exposure to infection and perceived risk of the infection, inadequate information on the pandemic and on the purpose and rules of quarantining (13, 43), the duration of quarantine (13, 15, 16, 44), dissatisfaction with the containment measures, in particular inadequate supplies of food, necessary goods, and medical availability (13, 15), quarantine-related stigmatization (45–47) and “frustration and boredom” due to confinement and reduced social and physical contact (13), and being associated with loneliness and financial losses due to quarantining (13). Perceived social support during quarantining has been a main protective factor (15, 48–50) as in most studies on pre- and peri-pandemic mental health (51).

**Figure 1:**
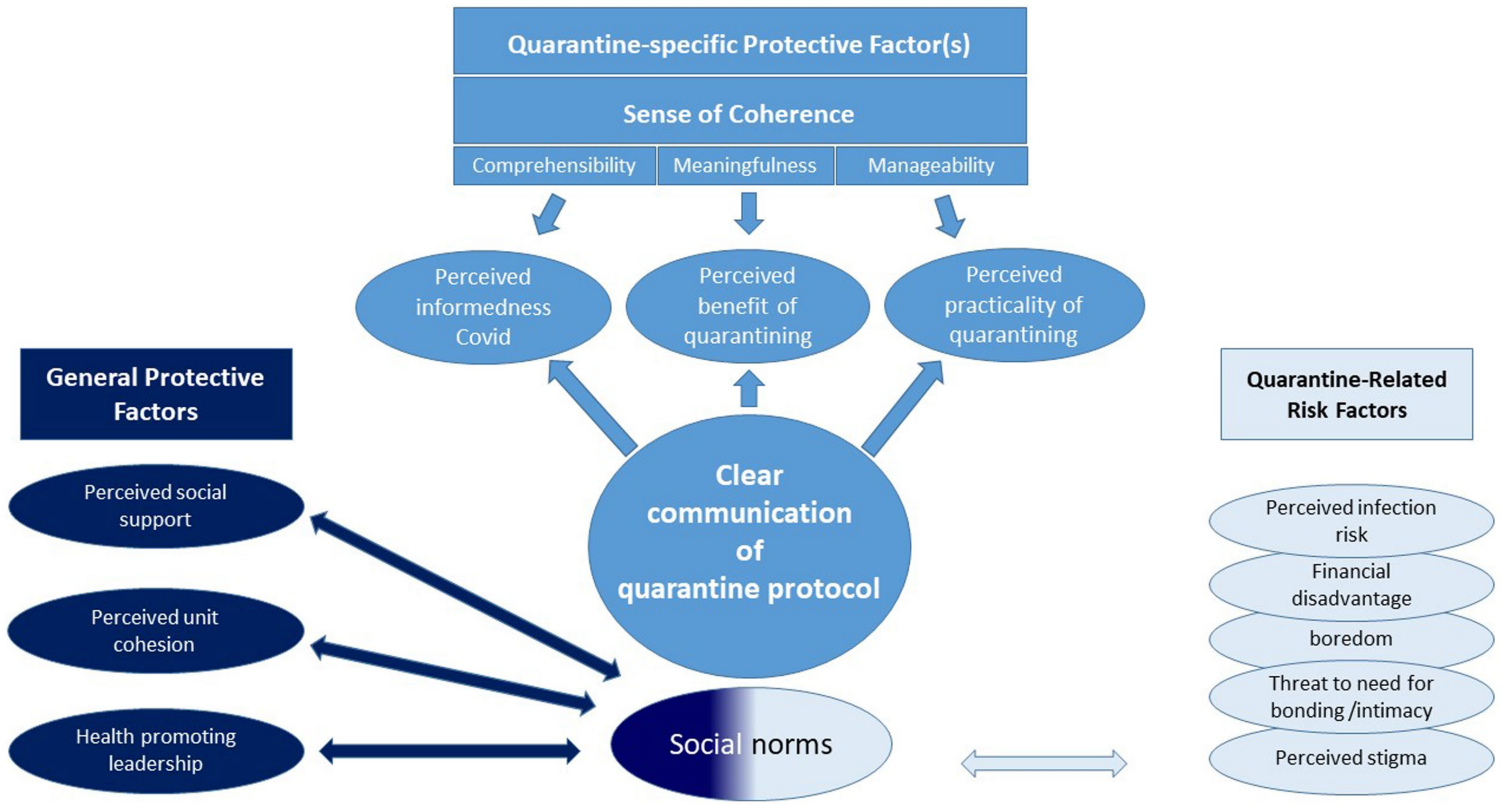
General and quarantine-specific risk and resilience factors which can be addressed by organizational health policy and military leadership (for definitions of the specific factors, please consult the glossary in Supplementary material 1).

Nonetheless, we conceptualize some of these perceived external factors in relation to two central overlapping psychological constructs, to situational meaningfulness (52) and to a situational sense of coherence with its three facets, namely, comprehensibility, meaningfulness, and manageability (52, 53) (see Figure 1). Situational comprehensibility is addressed as “feeling informed about COVID-19.” Situational meaningfulness is addressed as the “perceived benefit of quarantining” and situational manageability by “perceived practicalities of quarantining (perceived provision with food, medical care, daily supplies, contact, etc.).” The three facets can be addressed by “clear communication of the quarantine protocol” (see also Figure 1).

As for *social protective factors*, we are interested in different general and military-specific protective factors that have been found to be relevant in pre- and peri-pandemic studies on mental health (26, 51, 54, 55): general perceived social support (FSozu) and military-specific social support, including health-promoting leadership (27, 35), perceived unit cohesion (35, 56, 57), and quarantine conducive social norms of relevant others, here family and fellow soldiers (58).

Summing up, we are interested in the role of the following risk and resilience factors of mental health (see also Figure 1):

Questions 7 and 8: Which of the identified risk and resilience factors have the best predictive value for mental health at the end of post-deployment quarantine and for long-term mental health?

- *social protective factors* as perceived social support, perceived unit cohesion and health-promoting leadership, and quarantine conducive social norms of relevant others (family and fellow soldiers),
- a lower number respective lower values in quarantine-related risk factors, including financial disadvantages, perceived risk of infection, perceived stigma, boredom, and threat to the need for bonding/intimacy, and
- *quarantine-specific protective factors* relating to a situational sense of coherence and situational meaningfulness, including perceived informedness about COVID-19, clear communication of the quarantine protocol, perceived benefit of quarantining, and perceived practicality of quarantining.

In particular, we are interested if any of the quarantine-specific factors have a predictive value for post-deployment long-term mental health.

## 2 Methods

### 2.1 Recruitment procedure

Participants enrolled in the study at the in-processing into the pre-deployment quarantine facilities between February 2021 and March 2022. Administered informed consent was adapted to the quarantine protocol. PowerPoint presentations informed about the study as part of the in-processing at the quarantine facility. In addition, soldiers were informed by writing and provided phone numbers they could contact for further questions. Soldiers only participated in the study on a voluntary basis and upon prior written consent, including the consent on how to be contacted for the follow-up assessments after their return from the deployment. The study was conducted in line with the strict European and German data protection laws. The study has been approved by civilian and military commissions, including the Charité Ethics Commission (EA1/388/20).

No incentives have been provided for participating in the study. The last follow-up measurement took place in December 2022.

### 2.2 Design and measures

This study forms part of a prospective longitudinal design with up to five measurements, at the beginning and the end of pre- and post-deployment quarantine and 3 months after the soldiers’ return from the operational theater. The rationale for choosing assessment instruments was to reduce dropout by keeping the completion time as short as possible while at the same time relying on reliable and valid measures, when available as in the case of the Mini-SCL and the FSozU-K22. In the absence of validated scales assessing the relevant military- and pandemic-specific social support in the German language as well as the described quarantine-associated risk and resilience factors, the authors validated these scales with an independent subsample of 151 soldiers (36, 59) (Table 1).

**Table 1:** Assessments across the peri-pandemic deployment cycle.

Pre-deployment		Deployment	Post-deployment		
Pre-deployment quarantine			Post-deployment quarantine		Follow-up
T1	T2		T3	T4	T5

t1 and t2: beginning (t1) and end (t2) of pre-deployment quarantine. t3 and t4: beginning (t3) and end (t4) of post-deployment quarantine. t5: 3 months after completing the deployment.

#### 2.2.1 Mental health respective mental distress: brief symptom inventory-18 (Mini-SCL)

For the purpose of this study, we were less interested in diagnosing mental health disorders than in measuring the whole spectrum of mental health from mental wellbeing and mental distress as does the Brief Symptom Inventory (BSI), a shorter version of the Symptom Checklist (SCL-90) by Derogatis (60, 61). The German short version, BSI-18, has been renamed as the Mini-Symptom Checklist (Mini-SCL) (62). The General Severity Index score (GSI) of the Mini-SCL shows good reliability, with internal consistency (GSI α = 0.93), test–retest reliability (GSI *r_tt_* = 0.77), good convergent validity with PHQ depression (GSI *r* = 0.71, *p* < 0.0001), and PHQ anxiety (GSI *r* = 0.73, *p* < 0.0001). Psychiatric patients scored significantly higher on the GSI than two samples of non-patients (*χ^2^* = 775.21, *p* < 0.001) (63).

In the absence of pre-pandemic data of the cohort in point, the provision of age- and gender-specific norms (T-values) is necessary, which are provided for the GSI of the Mini-SCL (62).

#### 2.2.2 Perceived social support

Perceived social support is measured by a short version (K-22) of the “*Fragebogen zur Sozialen Unterstützung*” by Fydrich, Sommer, and Brähler (64), a widely used self-report measure for perceived social support in Germany. Reliability (Cronbach’s alpha) is excellent with α = 0.91, and retest reliability with a period of 2 months is good (*r* = 0.65). With respect to criterion validity, the scale is positively correlated with the size of one’s social network (*r* = 0.30, *p* < 0.01) and social competence (0.33 ≤ *r* ≤ 0.55, *p* < 0.01) and negatively correlated with loneliness (UCLA-L *r* = −0.77) and social stress (*r* = −0.54, *p* < 0.01). In respect to its predictive validity, it shows a number of negative correlations with mental health symptomatology, e.g., depression in a clinical sample (BDI: *r* = −0.52, *p* ≤ 0.001) and anxiety for the general population (STAI-G: *r* = −0.35, *p* < 0.01) (65). Healthy samples score higher on perceived social support than clinical samples [t(246.193) = 6.068; *p* ≤ 0.001, *d* = 0.52]. Norms for clinical and non-clinical groups are available (65, 66).

#### 2.2.3 Military- and pandemic-specific social support: perceived unit cohesion and health-promoting leadership

Scales for unit cohesion and military health-promoting leadership were developed, and 13 items were subjected to a main component analysis with varimax rotation (criterion eigenvalue >1) to assess the scales’ construct validity: This yielded three components, namely, unit cohesion, general health-promoting leadership (supervisor’s concern about the respective soldier’s health), and COVID-19-specific leadership, explaining 72.9% of the variance. In spite of good-to-excellent reliability (consistency) for the two scales and the four subscales (0.85 ≤ α ≤ 0.90), it is recommended only to use two or three scales based on the results for its construct validity (59). There is support for criterion validity, showing positive small correlations with perceived social support (FSozU-K22; *r* = 0.28, *p* < 0.001, 99% CI [0.066][0.469]), indicating that the scales are related, but still do measure different constructs (36, 59).

#### 2.2.4 Quarantine-associated risk and resilience factors

A total of 37 items were developed to capture nine quarantine-associated risk and resilience factors in the German language (59). Based on a separate subsample of 152 soldiers, 37 items were subjected to a main component analysis with varimax rotation, in which the nine conceptualized factors explained 59.23% of the variance (construct validity): “perceived knowledge about COVID-19,” “perceived benefit of the quarantine,” “perceived risk of infection (oneself, peers, relatives),” “perceived practicality of the quarantine” (supply of food, medical care, information, and loved ones are cared for), “positive social norms toward the quarantine by relevant others (military peers and family)” [short: social norms], “perceived stigmatization” (by fellow soldiers/peers) and “boredom,” “perceived clarity of communication concerning the quarantine protocol” (purpose, duration, and rules relating to isolation), and “fulfilled need for intimacy/bonding.” Seven factors yielded satisfactory to good reliability (consistency; 0.73 ≤ α ≤ 0.83), while the two most heterogeneous scales, namely, “fulfilled need for intimacy/bonding” and “clear communication of the quarantine protocol” (α ≤ 0.58), had to be z-standardized to reach satisfactory consistency (α > 0.7): For consistency reasons, all items were z-standardized before calculating scale means. More details on the scales and their validation have been reported previously (36, 59).

*Financial disadvantage due to the quarantine* was captured by one item: “due to the quarantine, I am/my family is experiencing financial disadvantages (e.g., additional costs for childcare, shortened deployment, etc.).”

*Assessment of duration of the COVID-19 pandemic:* Participants filled in the date they answered the respective questionnaire. The date was coded as year-month-day.

### 2.3 Analysis

Most of the analyses were carried out in SPSS 29, including correlations, multiple regression analysis, and ANOVA with repeated measures. When not available in SPSS 29, effect sizes and confidence intervals (CIs) were calculated manually with the help of two websites: https://www.psychometrica.de/korrelation.html for correlations (62) and https://effect-size-calculator.herokuapp.com/ for partial eta squared (63) and omega squared (64).

With the purpose of identifying mental health trajectories, exploratory analyses were conducted using R statistical software version 4.0.5 (67) on a Windows operating system. Exploratory data analysis was performed using visualizations created with *tidyverse packages* (68). Descriptive statistics, including means, medians, and standard deviations, were computed using the *DescrTab2 package* (69) for the exploratory interpretation of the study results.

### 2.4 Required sample size

We adjusted the alpha error for multiple tests taking into account a previous publication on the course of pre-deployment quarantine (36). Required sample sizes were calculated with the help of GPower (61). A minimum sample size of 94 participants is mandatory to detect changes with an effect size of *f* = 0.15 across pre- and post-deployment quarantine, and a sample size of 113 is required to detect a potential delayed onset of mental health symptoms 3 months post-deployment.

As for predicting mental health at the end of post-deployment quarantine and 3 months post-deployment, the *a priori* computed required sample size is 143 for detecting an effect of *f*^2^ = 0.15 and a sample size of 76 is required for an effect size of at least *f*^2^ = 0.35. For more details, please refer to Supplementary material 2.

## 3 Results

### 3.1 The sample and accounting for potential bias

#### 3.1.1 The sample

The sample: From 928 soldiers who enrolled in the study at the beginning of pre-deployment quarantine (t1), 907 also participated at the end of pre-deployment quarantine (t2), 143 at the beginning of post-deployment quarantine (t3), 132 at the end of post-deployment quarantine (t4), and 308 3 months after returning home (t5).

Reduced numbers of participation during post-deployment quarantine were mainly due to its early suspension (depending on the respective operational theater). This is reflected in 84% (of 143 quarantinees) reporting on post-deployment quarantine in 2021 and 16% (of 143) in 2022 and in more accumulated quarantining experience 3 months post-deployment for soldiers reporting on post-deployment quarantine (see Supplementary material 3).

For a description of the sample at the beginning of pre-deployment quarantine (t1) and approximately 8 months later, 3 months post-deployment (t5), please refer to Table 2.

**Table 2:** Sample at the beginning of pre-deployment quarantine (t1) and at follow-up approximately 8 months later, 3 months post-deployment (t5).

Sample	T1	T5
Age (years)		
Minimum – maximum *M/Mdn*	18–64 36 / 34	18–64 37.75 / 36
Sex	87.5%: 12.5%	88.2%: 11.8%
Rank (socio-economic status)		
enlisted personnel/private/corporal non-commissioned officers commissioned officers	10.7% 54% 35.3%	7.7% 52.2% 39.7%
Partnership	70%	81.8%
Children	42.5%	54.2%
Single caretaker	1.9%	2.6%
Children in emergency-care	8.9%	9.7%
Deployment experience		
Number of missions abroad *Min – max* *Mdn / M / SD* Accumulated days deployed *Min – max* *Mdn / M / SD*	0–60 1 / 2.59 / 4.08 0–2,465 140 / 236 / 310	0–40 2 / 3.13 / 4.34 0–2,465 160/ 291 / 381
Quarantining experience		
No previous experience Previous experience (days: *Mdn / M / SD*)	25% 14 / 13.62 / 21.57	0% 20 / 25 / 19.79

#### 3.1.2 Accounting for potential bias or limitations caused by dropout

Potentially biased results due to dropout or missing data are accounted for in three ways: (1) It was analyzed if missing data were related to any of the outcome variables and the predictors, the military- and quarantine-specific risk and resilience factors (predictors), and sociodemographic variables (see Supplementary material 3). (2) We aimed to maintain as much of the sample size as possible, as a result of which we refrained from using sociodemographic variables as covariates except for following up on significant sociodemographic effects during pre-deployment quarantining in a previous analysis (36, 70). (3) The potential impact of “suspended post-deployment quarantine respective missing data” on mental health 3 months post-deployment was accounted for by introducing the between factor “suspended post-deployment quarantine/missing data” into the ANOVAs with repeated measures.

*Correlations with dropout for t5 and suspended post-deployment quarantine/non-participation in the study during post-deployment quarantine:* Dropout is not directly related to any of the psychosocial outcome variables during pre-deployment quarantine (|0.016| ≤ *r*s ≥ |0.022|, 0.431 ≤ *p*s ≥ 0.564) nor is the suspended post-deployment quarantine or the non-participation in the study during post-deployment quarantine related to any of the psychosocial outcome variables (|0.010| ≤ *r*s ≥ |0.084|, 0.076 ≤ *p*s ≥ 0.830; for further information, please refer to Supplementary material 3).

### 3.2 Is there a delayed deterioration of mental health or its protective factors, perceived unit cohesion, and perceived social support 3 months post-deployment?

Three one-way repeated measures ANOVAs were conducted with the dependent variables mental health (Mini-SCL), perceived social support, and perceived unit cohesion—and the between factor “suspended post-deployment quarantine/missing.”

**Figure 2:**
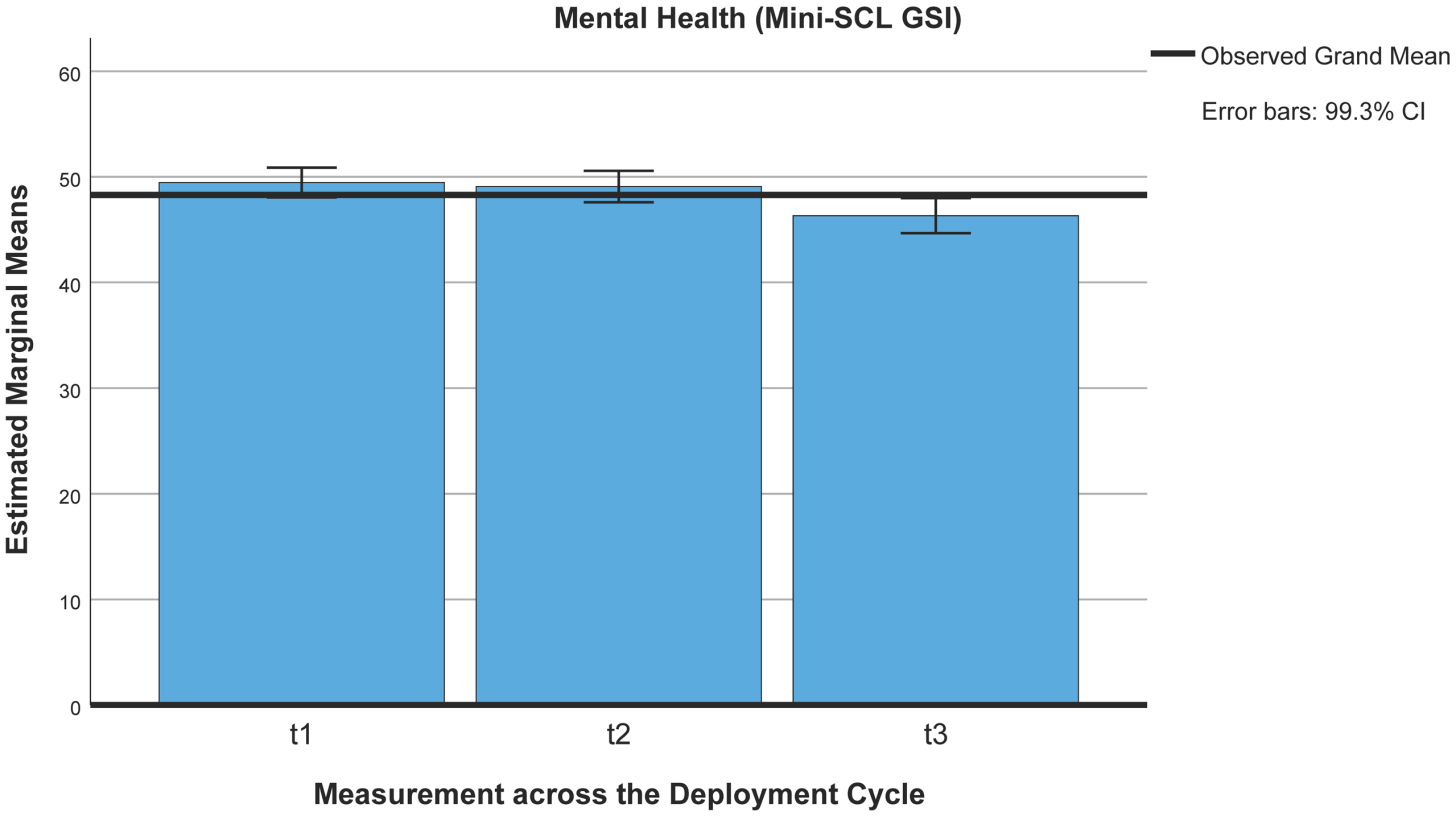
Mental health (Mini-SCL/GSI) across the deployment cycle: pre-deployment quarantine and 3 months post-deployment. t1 = beginning of pre-deployment quarantine, t2 = end of pre-deployment quarantine, t5 = 3 months post-deployment.

Using Pillai’s trace, the ANOVA yielded a significant improvement for mental health (Mini-SCL T-values) between pre-deployment quarantine and 3 months post-deployment [*V* = 0.140, *F*(2,265) = 21.54, *p* < 0.001, η^2^ = 0.140, 99% CI [0.051, 0.239], ω^2^ = 0.13, *M*_t1_ = 49.57, *SE*_t1_ = 0.54, *M*_t2_ = 49.14, *SE*_t2_ = 0.57, *M*_t5_ = 46.32, *SE*_t5_ = 0.61, *n* = 268 (*p* < 0.001)] but no difference across pre-deployment quarantine (*p* = 1; see Figure 2).

Following up on the interaction effect for accumulated previous quarantining experience (36), no interaction effect was found for the accumulated experience of quarantining before pre-deployment quarantine [*V* = 0.018, *F*(2,182) = 1.679, *p* = 0.189, η^2^ = 0.018, 99% CI [0, 0.009], ω^2^ = 0.018] nor for the accumulated quarantining experience 3 months post-deployment [*V* = 0.006, *F*(2,213) = 0.657, *p* = 0.520, η^2^ = 0.006, 99% CI = [0, 0.043], ω^2^ = 0].

**Figure 3:**
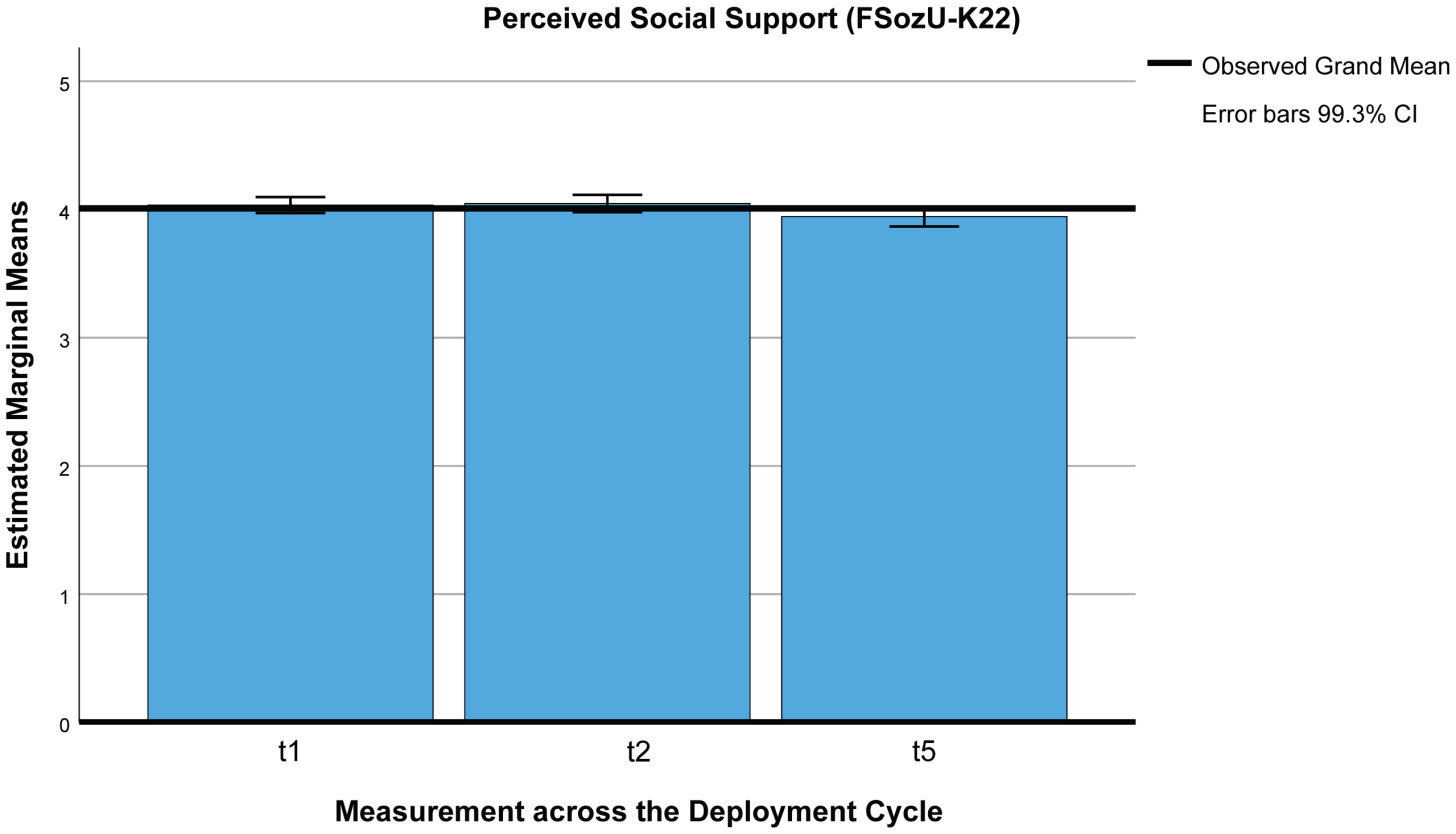
Perceived social support across the deployment cycle: pre-deployment quarantine and 3 months post-deployment. t1 = beginning of pre-deployment quarantine, t2 = end of pre-deployment quarantine, t5 = 3 months post-deployment.

Social support (FSozU-K22) was perceived to be slightly lower 3 months post-deployment than during pre-deployment quarantine [*M*_t1_ = 4.03, *SE*_t1_ = 0.02, *M*_t2_ = 4.05, *SE*_t2_ = 0.03, *M*_t5_ = 3.94, *SE*_t5_ = 0.03, *n* = 268 (*p*s < 0.001)] but did not differ across pre-deployment quarantine (*p* = 1), using Pillai’s trace [*V* = 0.112, *F*(2,266) = 16.85, *p* < 0.001, η^2^ = 0.11, 99% CI [0.033, 0.207], ω^2^ = 0.11; see Figure 3].

Following up on the interaction effect for age (36), no interaction effect was found for age [*V* = 0.018, *F*(2,182) = 1.679, *p* = 0.189, η^2^ = 0.018, 99% CI [0, 0.089], ω^2^ = 0.01] nor for the accumulated quarantining experience 3 months post-deployment [*V* = 0.006, *F*(2,213) = 0.657, *p* = 0.520, η^2^ = 0.006, 99% CI [0, 0.052], ω^2^ = 0].

Contrary to expectations, perceived unit cohesion did not change at all [Pillai’s trace, *V* = 0.004, *F*(2,264) = 0.482, *p* = 0.618, η^2^ = 0.004, 99% CI [0, 0.039], ω^2^ = 0].

**Figure 4:**
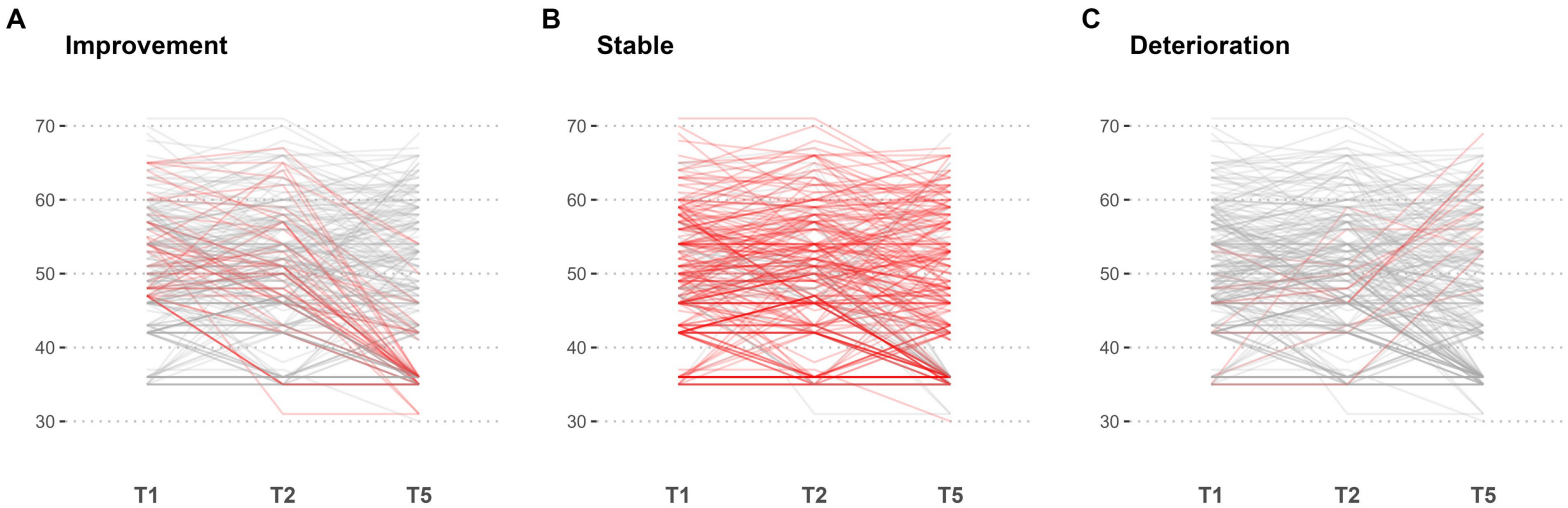
Individual level line plot of all patients for Mini-SCL (GSI). Each line gives the trajectory of an individual over time, with the x-axis representing the points in time t1, t2, and t5, and the y-axis representing the corresponding values. The lines are color-coded to differentiate between groups: improvement **(A)**, stable **(B)**, and deterioriation **(C)**, with the highlighted group plotted in red and all other trajectories coloured in grey.

**Figure 5:**
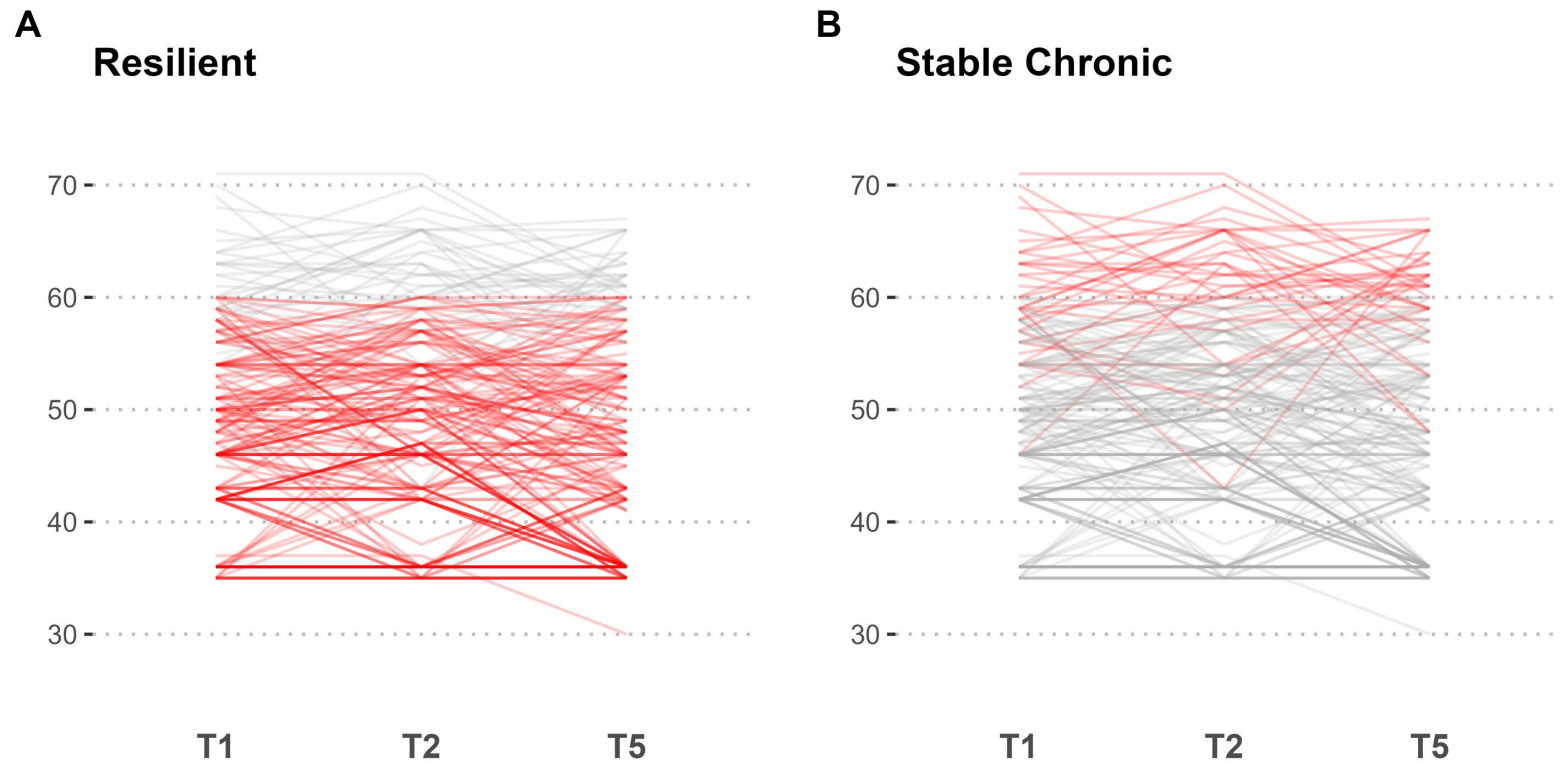
Subgroup analysis of patients with stable trajectories for Mini-SCL (GSI). Points in time T1, T2, and T5 are indicated on the x-axis with corresponding values on the y-axis. Out of all patients identified as stable, two subgroups are highlighted in red: high chronic stable **(A)** and resilient **(B)**, with other trajectories coloured in grey, respectively.

Mental health trajectories were identified in a two-step exploratory analysis: In the first step, three trajectories were identified: A stable trajectory was defined as one that does not change more than one SD over time, an improvement in mental health was defined as a decrease in mental health symptomatology by more than one SD over time, a deterioritaion in mental health was defined in mental health symptomatology (GSI) was defined as a change of more than one SD over time. These resulted in the following trajectories (see Figure 4; Table 3): 79.2% were identified as stable, for 12.6% the GSI decreased post-deployment (adaptation), and for 4.6% the GSI increased. Based on qualitative clinical evaluations of the range of T-values for the stable trajectory between approximately 35 and over 70 (see Tables 3, 4), it was decided to divide the stable trajectories into two: a resilient-stable trajectory, permanently staying below a T-value of 60, and a “chronic-stable” trajectory, for which T-values exceed 60 at least at one of the three assessments. Based on these criteria, 66.6% were identified as “resilient-stable” and 12.6% as “chronic-stable” (see Figure 5; Table 4). Descriptive characterizations of the four trajectories by sociodemographic variables and risk and resilience factors can be found in the Supplementary material 4.

**Table 3:** Statistic description of mental health trajectories: “stable”, “improving” and “deteriorating”.

	Improvement (*n* = 49)	Deterioration (*n* = 14)	Stable (*n* = 239)
T1			
Count	49	14	239
Mean (*SD*)	53.388 (5.926)	44.571 (6.198)	48.787 (8.825)
Median	53.000	46.000	49.000
Q1,Q3	48.000, 57.000	42.000, 48.000	42.000, 55.500
T2			
Count	44	12	212
Missing data	5	2	27
Mean (*SD*)	50.318 (8.672)	47.500 (6.303)	48.915 (9.137)
Median	51.000	47.000	49.000
Q1,Q3	46.000, 56.250	45.250, 50.250	42.000, 56.000
T5			
Count	49	14	239
Mean (SD)	37.633 (5.122)	59.143 (5.696)	47.314 (9.775)
Median	36.000	59.000	47.000
Q1,Q3	35.000, 36.000	56.000, 63.500	36.000, 55.500

**Table 4:** Statistic description for mental health trajectories: “resilient stable” and “chronic stable”.

	Stable chronic (*n* = 38)	Stable resilient (*n* = 201)
T1		
Count	38	201
Mean (*SD*)	60.763 (5.654)	46.522 (7.372)
Median	60.000	46.000
Q1,Q3	57.250, 64.000	42.000, 52.000
T2		
Count	35	177
Missing	3	24
Mean (*SD*)	61.114 (5.974)	46.503 (7.599)
Median	62.000	47.000
Q1,Q3	58.500, 66.000	42.000, 53.000
T5		
Count	38	201
Mean (*SD*)	61.000 (4.685)	44.726 (8.205)
Median	62.000	45.000
Q1,Q3	59.000, 64.000	36.000, 52.000

### 3.3 Predicting mental health 3 months post-deployment based on pre-deployment quarantine

A significant regression equation was found predicting mental health 3 months post-deployment (t5) [*F*(4,261) = 22.26, *p* < 0.001] by predictors assessed at the end of pre-deployment quarantine (t2). Four predictors explained 24% of the variance (*R* = 0.50, *R*^2^ = 0.25, corrected *R*^2^ = 0.24, 99% CI [0.14, 0.35], ω^2^ = 0.24): clear communication of the quarantine protocol [Δ*R*^2^ = 0.12, *F*(1,264) = 35.42, *p* < 0.001, 99% CI [0.04, 0.22], ω^2^ = 0.12], perceived unit cohesion [Δ*R*^2^ = 0.06, *F*(1,263) = 20.02, *p* < 0.001, 99% CI [0.01, 0.16], ω^2^ = 0.07], fulfilled need for intimacy/bonding [Δ*R*^2^ = 0.05, *F*(1,262) = 16.25, *p* = 0.003, 99% CI [0.01, 0.11], ω^2^ = 0.05], and perceived social support (FSozU-K22), [Δ*R*^2^ = 0.05, *F*(1,261) = 9.07, *p* < 0.001, 99% CI [0.00, 0.11], ω^2^ = 0.03]. The robustness of the model was confirmed when all identified predictors were re-entered in a regression analysis applying bootstrapping (see Table 5).

**Table 5:** Predicting mental health 3 months post-deployment by risk and resilience factors assessed at the end of pre-deployment quarantine.

		B	*SE*	Beta	*t*	*p*	LL CI_98%_	UL CI_98%_	*r(zero order)*
1	(Constant)	0.012	0.057		0.210	0.834	−0.136	0.160	
	zt2_clear_quarantine_protocol	−0.581	0.096	−0.348	−6.061	<0.001	−0.830	−0.332	−0.348
2	(Constant)	−0.002	0.055		−0.042	0.967	−0.146	0.141	
	zt2_clear_quarantine_protocol	−0.467	0.097	−0.280	−4.837	<0.001	−0.718	−0.217	−0.348
	zt2_Unit_Cohesion	−0.311	0.072	−0.249	−4.308	<0.001	−0.498	−0.124	−0.326
3	(Constant)	0.012	0.054		0.217	0.828	−0.128	0.151	
	zt2_clear_quarantine_protocol	−0.339	0.099	−0.203	−3.416	<0.001	−0.596	−0.081	−0.348
	zt2_Unit_Cohesion	−0.317	0.070	−0.254	−4.520	<0.001	−0.499	−0.135	−0.326
	zt2_intimacy_bonding	−0.330	0.082	−0.231	−4.032	<0.001	−0.543	−0.118	−0.315
4	(Constant)	0.011	0.053		0.207	0.836	−0.127	0.149	
	zt2_clear_quarantine_protocol	−0.293	0.099	−0.175	−2.960	0.003	−0.549	−0.036	−0.348
	zt2_Unit_Cohesion	−0.265	0.071	−0.213	−3.724	<0.001	−0.450	−0.080	−0.326
	zt2_intimacy_bonding	−0.346	0.081	−0.242	−4.283	<0.001	−0.556	−0.136	−0.315
	zt2_FSozU	−0.291	0.096	−0.171	−3.036	0.003	−0.539	−0.042	−0.269

Linear model of predictors, with 98% BCa CI. CI and SE based on 2,000 bootstrap samples. *ΔR^2^* = 0.12 for Step 1. *ΔR^2^* = 0.06 for Step 2. *ΔR^2^* = 0.05 for Step 3. (*ps* < 0.001). *ΔR^2^* = 0.03 for Step 4 (*p* = 0.003). F(4,263) = 22.23, *p* < 0.001, *R* = 0.50, *R^2^* = 0.25, corrected *R^2^* = 0.24, 99% CI [0.13, 0.35], *ω^2^* = 0.24. ^a^Dependent variable: mental health 3 months post-deployment (Mini-SCL at t5 based on z-standardized age- and gender-specific T-values). ^b^Predictors at the end of pre-deployment quarantine (z-standardized): (1) clear communication of the quarantine protocol, (2) perceived unit cohesion, (3) fulfilled need for intimacy/bonding, and (4) perceived social support (FSozU K22).

After Bonferroni corrections, none of the sociodemographic variables correlated significantly with mental health 3 months post-deployment (0.021 ≤ *p*s ≤ 0.479; sign. one-tailed). No regression equation was calculated using SPSS 29.

### 3.4 Different approaches to pre- and post-deployment quarantining

#### 3.4.1 Changes in psychosocial wellbeing across pre- and post-deployment quarantining

Three one-way repeated measures ANOVAs were conducted with the dependent variables mental health, perceived social support, perceived unit cohesion, and quarantine adherence.

**Figure 6:**
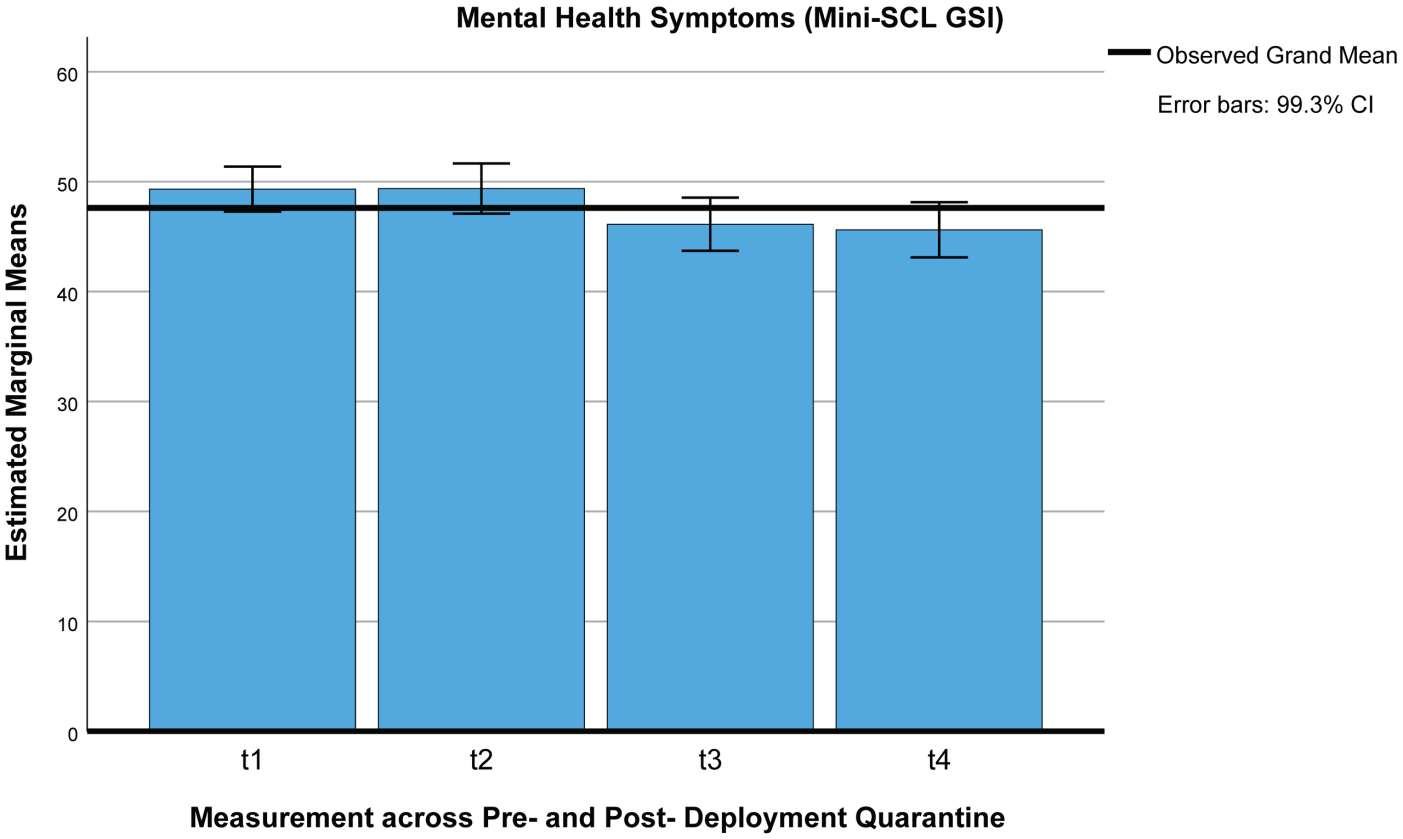
Mental health (Mini-SCL/GSI) across pre- and post-deployment quarantine pre-deployment quarantine: t1 = beginning, t2 = end; post-deployment quarantine: t3 = beginning, t4 = end.

Using Pillai’s trace, the ANOVA yielded a significant difference for mental health [Mini-SCL; *V* = 0.185, *F*(3,110) = 8.34, *p* < 0.001, η^2^ = 0.185, 99% CI [0.03, 0.33], ω^2^ = 0.16], displaying significant differences between pre- and post-deployment quarantine (3.2 ≤ *M*_Diff_ i-j_ ≤ 3.7, *p*s < 0.001) but there was no difference between the start and the end of pre- or post-deployment quarantining (*M*_t1-t2_ = −0.05, *M*_t3-t4_ = 0.50, *p* = 1), with an improvement of mental health during post-deployment quarantining as compared to pre-deployment quarantining [*M*_t1_ = 49.32, *SE*_t1_ = 0.75, *M*_t2_ = 49.37, *SE*_t2_ = 0.83, *M*_t3_ = 46.12, *SE*_t3_ = 0.88, *M*_t4_ = 45.62, *SE*_t4_ = 0.91, *n* = 113; see Figure 6].

**Figure 7:**
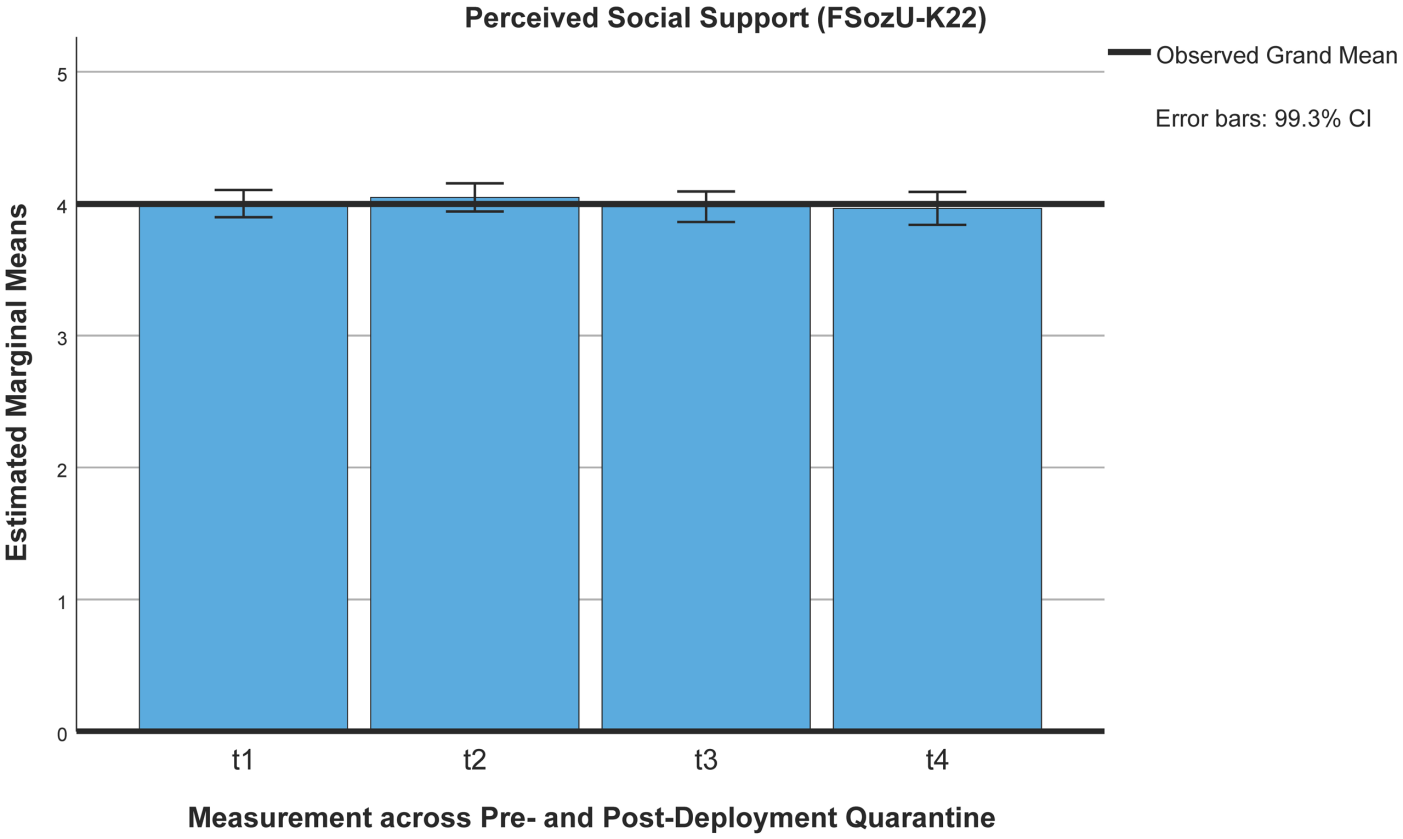
Perceived social support (FSozU-K22) across pre- and post-deployment quarantine pre-deployment quarantine: t1 = beginning, t2 = end; post-deployment quarantine: t3 = beginning, t4 = end.

Using Pillai’s trace, the ANOVA yielded a significant effect for perceived social support [*V* = 0.11, *F*(3,110) = 4.62, *p* = 0.004, η^2^ = 0.11, 99% CI [0, 0.25], ω^2^ = 0.09; see Figure 2]. After Bonferroni corrections, the only significant decrease in perceived social support was between the end of pre-deployment (t2) and the end of post-deployment (t4) quarantining (*M*_t2_ = 4.05, *SE*_t2_ = 0.04, *M*_t4_ = 3.96, *SE*_t4_ = 0.05, *p* = 0.005), while the perceived decrease in social support between the end of pre-deployment quarantining (t2) and the start of post-deployment quarantining lost significance (*M*_t3_ = 3.98, *SE*_t3_ = 0.04, *p* = 0.009). Perceived social support at the start of pre-deployment quarantine (*M*_t1_ = 4.00, *SE*_t1_ = 0.04) did not differ from any of the other points of measurement across quarantining (0.37 ≤ *p* ≤ 1; Figure 7).

Contrary to our expectations, perceived unit cohesion did not change across pre- and post-deployment quarantine, according to Pillai’s trace [*V* = 0.006, *F*(3,105) = 0.219, *p* = 0.883, η^2^ = 0.01, 99% CI [0, 0.06], ω^2^ = 0].

#### 3.4.2 Predicting mental health at the end of post-deployment quarantine

A significant regression equation was found predicting mental health at the end of post-deployment quarantine [*F*(2,99) = 22.22, *p* < 0.001] by predictors assessed at the beginning of post-deployment quarantine. Two predictors explained 30% of the variance [*R* = 0.56, *R*^2^ = 0.31, corrected *R*^2^ = 0.30, 98% CI [0.12, 0.47], ω^2^ = 0.30]: perceived stigma [Δ*R*^2^ = 0.25, *F*(1,100) = 32.92, *p* < 0.001, 98% CI [0.08, 0.41], ω^2^ = 0.24] and clear communication of the quarantine protocol [Δ*R*^2^ = 0.06, *F*(1,99) = 8.91, *p* = 0.004, 98% CI [0.00, 0.24], ω^2^ = 0.07]. The robustness of the model was confirmed by entering the predictors identified in a regression analysis applying bootstrapping (see Table 6).

**Table 6:** Predicting mental health at the end of post-deployment quarantine.

	B	*SE*	Beta	*t*	*p*	LL CI_98%_	UL CI_98%_	*r(zero order)*	
1	(Constant)	0.040	0.086		0.460	0.646	−0.187	0.266	
	t3 z_stigma	−0.655	0.119	−0.479	−5.484	<0.001	−0.969	−0.342	−0.479
2	(Constant)	0.036	0.083		0.436	0.664	−0.182	0.254	
	t3 z_stigma	−0.599	0.116	−0.438	−5.147	<0.001	−0.905	−0.294	−0.479
	t3 z_clear quarantine protocol	−0.412	0.137	−0.257	−3.018	0.003	−0.771	−0.054	−0.327

The linear model of predictors with 98% BCa CI. CI and *SE* based on 2,000 bootstrap samples. *ΔR^2^* = 0.23 for Step 1. *ΔR^2^* = 0.06 for Step 2 (*ps* < 0.001). F(2,100) = 20.79, *p* < 0.001, *R* = 0.55, *R^2^* = 0.29, corrected *R^2^* = 0.28, 98% CI [0.03, 0.34], *ω^2^* = 0.16. ^a^Dependent variable: Mini-SCL at the end of post-deployment quarantine (based on z-standardized age- and gender-specific T-values). ^b^Predictors at the beginning of post-deployment quarantine: perceived/expected stigmatization by fellow soldiers and clear communication of the quarantine protocol.

None of the correlations for sociodemographic variables with mental health remained significant after Bonferroni correction (α = 0.01): age (*r* = 0.22, *p* = 0.015), sex (*r* = 0.19, *p* = 0.026), and rank (*r* = 0.20, *p* = 0.022, *n* = 102). When adjusting the alpha error at α = 0.01, no stepwise regression was computed. Without Bonferroni corrections, the only sociodemographic variable predicting mental health at the end of post-deployment quarantine was age (*R* = 0.22, *R*^2^ = 0.05, corrected *R*^2^ = 0.04, *p* = 0.03, 98% CI [0.42, 0.53], ω^2^ = 0.05).

### 3.5 Duration of the pandemic and psychosocial wellbeing

As for exploring the relationship between pandemic duration and psychosocial wellbeing, we did not find any significant correlations between the duration of the pandemic on the one hand and perceived unit cohesion (*r*_t1_ = −0.054, *p* = 0.054, *N* = 880; *r*_t2_ = −0.056, *p* = 0.056, *N* = 791; *r*_t5_ = 0.010, *p* = 0.433, *N* = 303) or perceived social support (*r*_t1_ = −0.035, *p* = 0.147, *N* = 910; *r*_t2_ = −0.069, *p* = 0.027, *N* = 789; *r*_t5_ = 0.002, *p* = 0.484, *N* = 299) on the other hand. However, the duration of the pandemic showed very small significant positive correlations with mental health symptoms over time (*r*_t1_ = 0.092, *p* = 0.003, *N* = 905; *r*_t2_ = 0.115, *p* < 0.001, *N* = 791; *r*_t3_ = 0.161, *p* = 0.003, *N* = 300).

## 4 Discussion

Mandatory pre- and post-deployment quarantining added further constraints on soldiers deployed abroad during the pandemic. Contrary to previous research on civilian quarantining, no adverse short-term psychosocial effects of quarantining were identified. Therefore, we were interested if there was a delayed deterioration of psychosocial wellbeing post-deployment, a potentially different impact of post-deployment quarantine, and quarantine-associated risk and resilience factors predicting mental health post-deployment. Instead of a deterioration of general psychosocial wellbeing post-deployment, mental health symptoms were further reduced, and perceived unit cohesion remained stable across pre- and post-deployment quarantine and at the 3-month follow-up. Only perceived social support minimally decreased at a 3-month follow-up post-deployment, while perceived social support also did not differ significantly between pre- and post-deployment quarantine or across quarantining. Although we did not find an adverse impact of deployment-related quarantining on mental health, we found a minimal but significant increase in mental health symptoms with ongoing duration of the pandemic pre-deployment and at a 3-month follow-up, in spite of expected seasonal variations depending on general infection rates and containment measures (71–74).

In a descriptive exploratory analysis of mental health trajectories across the deployment cycle, we identified four trajectories based on clinical criteria (changes of at least one standard deviation, respectively, exceeding a T-value of 60): 66.6% with a stable resilient trajectory, 16.2% with decreasing mental health symptomatology, 4.6% with increasing mental health symptoms (delayed onset), and 12.6% with a stable chronic trajectory (above a T-value of 60). These four resilience trajectories are in line with pre- and peri-pandemic research based on large civilian samples (25, 26), while the only study on mental health trajectories across the deployment cycle suggests one more trajectory, a “chronic-stable” trajectory (21). While we cannot attribute any of the resilience trajectories to the impact of quarantining, the different mental health trajectories across the deployment cycle indicate that approximately 12.6% of deployed soldiers are at risk, and 4.6% of soldiers showed a delayed onset of mental health symptoms only 3 months post-deployment. However, not everyone who displays mental distress before deployment does so 3 months post-deployment, as is the case for 16.2% (adaptation). Given the study’s exploratory nature, these results are primarily to be interpreted descriptively.

In total, 24% of mental health at follow-up 3 months post-deployment are predicted by a mix of quarantine-specific, military-specific, and general resilience factors assessed approximately 7 months earlier: clear communication of the quarantine protocol (12%), perceived unit cohesion (an additional 6%), fulfilled need for intimacy/bonding during quarantine (an additional 5%), and perceived social support (FSozU-K22; an additional 3%). While just 24% of post-deployment mental health is explained by resilience factors assessed 7 months earlier, the relatively strong role of clear communication of the quarantine protocol for long-term mental health is striking. While we did not find evidence for an overall adverse impact of quarantining on psychosocial wellbeing, 30% of the variance in mental health at the end of post-deployment quarantine was only predicted by quarantine-specific factors: perceived stigma and clear communication of the quarantine protocol when entering post-deployment quarantine.

These results indicate that pre-deployment quarantining and post-deployment quarantining were not as harmful as initially expected, while the duration of the overall pandemic might have minimally impacted mental distress. Some caution regarding delayed mental health effects is given due to a minimal decrease in perceived social support. The decrease in perceived social support was not related to overall mental health. At the same time, the effect might be the reverse: Higher perceived social support might reflect the exceptional full-time care conditions during pre-deployment quarantining and the decrease reflecting a return to the “normal” level. However, a decrease in perceived social support can also precede heightened mental distress. While the identified chronic-stable trajectory and delayed onset of mental health cannot be attributed to quarantining, one has to assume that 17.2% of deployed soldiers experience heightened mental distress post-deployment, which is in the range of pre-pandemic prevalence rates for any mental disorder post-deployment for a sample of 1,439 German deployed military service members (16.6, 95% CI 14.6–18.9) (20). In a US-American study with almost 8,000 military service members, trajectories have been associated with loneliness and deployment-related stress factors than with combat (21). Identifying the relevant risk and resilience factors predicting these trajectories for German deployed soldiers would facilitate earlier identification of the relevant groups at risk.

Higher mental distress at the beginning of pre-deployment quarantining might also be due to tense anticipation of the quarantining situation and the deployment as well as organizational stress cumulating before deploying. The research results are also in line with two previous studies on quarantining with civilian travelers reporting that the purpose (75) and the conditions of quarantining (76) can make a difference. In a Canadian cross-sectional study, people quarantined due to traveling did not experience increased self-harm or suicidal ideations as opposed to other quarantinees (75). During a hotel-based quarantine for 533 South Australian travelers, the returnees’ mental distress slightly decreased under favorable quarantining conditions, including the provision of daily goods, medical and psychological support, and clear information on the quarantine protocol. This result indicates that favorable quarantining conditions can compensate stress caused by quarantining, at least if quarantining is due to traveling (76). The higher stress level at the inprocessing into the quarantine facility might be attributed to initial tense anticipations. These studies have also in common that quarantine was not related to a previous infection or contact with an infected person, which—when perceived as life-threatening—can result in trauma-related mental distress. Perceived infection risk was identified as a relevant risk factor in quarantining and isolation (13, 43). This risk factor, perceived infection risk for oneself as well as for relevant others (fellow soldiers and family), remained relatively low (−0.78 ≤ M ≤ −0.21, 0.11 ≤ *SE* ≤ 0.13) for our study group, while general risk was perceived to be high across all points of measurements (0.81 ≤ 1.3, 0.09 ≤ *SE* ≤ 0.12, on a scale of −2 to +2; see Figure in Supplementary material 6). The military quarantinees in our study were also fully paid during quarantining, thereby not facing (severe) financial disadvantages or even existential financial threats by quarantining, which is reflected in equally low evaluations of quarantine-associated financial disadvantages across pre- and post-deployment quarantining (−1.7 ≤ *M*_t1_-*M*_t4_ ≤ −1.6, 0.03 ≤ *SE* ≤ 0.08, *p* = 0.61, on a scale from −2 to +2; see Figure in Supplementary material 7).

### 4.1 Strengths and limitations

Planned quarantining allowed us to implement a prospective research design starting with an assessment at the beginning of the quarantine, while due to legal and ethical constraints, no control group could be implemented. This regulated quarantining allowed us to study close to ideal quarantining conditions as recommended by reviews and meta-analyses (13–16) as a number of quarantine-related risk and resilience factors were controlled for, including the absence of infection-related traumatic experience, the practicalities, including the provision of daily needs, medical care and a 24/7 hotline, and potential financial disadvantages. These could be complemented by manipulation checks, showing low perceived personal infection risk and very low quarantine-related financial disadvantages (see Supplementary materials 6, 7).

As this study focused on active military service personnel, the sample’s sociodemographics were typical for deploying military personnel with a majority of male soldiers (about 90%) and a minority of 10% female soldiers and an age range between 18 and 64 years. Therefore, limitations to the generalization of results apply to children, adolescents, and people at retirement age. The results might be less representative for women in the overall German population but are quite representative for the Bundeswehr, the respective German deployed military personnel.

During pre-deployment quarantine, the recruited sample of 928 soldiers was close to being representative with respect to sociodemographic variables for the German deploying troops with a low number of missing data. However, the early suspension of post-deployment quarantine resulted in substantial missing data of approximately 85% for post-deployment quarantine (t3 and t4). Seven months later, 3 months post-deployment, approximately a third of the initial sample participated in the study resulting in missing values up to 70%. A strong limitation is that we cannot disentangle the factors “suspended post-deployment quarantine” and “missing data” for post-deployment quarantine. However, we controlled for a differential mental health impact by employing the between factor “suspended quarantine/missing” in the ANOVA with repeated measures. In a separate study, in which we merged peri-pandemic data from a subsample, which had undergone pre- and post-deployment quarantine, and pre-pandemic data, mental health improved already during post-deployment quarantine and remained stable 3 months post-deployment (70), which supports the conclusions in this study.

Dropout 3 months post-deployment was not related to the psychosocial outcome variables but showed small correlations with sociodemographic variables and two quarantine-associated variables, showing that soldiers of younger age, lower rank, and with less deployment experience were underrepresented 3 months post-deployment, with younger age being the main variable. Soldiers with less perceived infection risk and more boredom were slightly more likely to drop out 3 months post-deployment. Therefore, caution should be applied against premature generalizations, in particular for soldiers of younger age and lower rank.

Dropout respective missing data and decreased sample size did not allow us to test the best model fit for a different number of trajectories or predict trajectories based on sociodemographic variables or risk and resilience factors. The results are primarily to be interpreted descriptively, focusing on summarizing and understanding the patterns and relationships within the dataset. The findings provide initial insights and are intended to generate hypotheses for further investigation. However, to our knowledge, this is the first descriptive analysis of mental health trajectories across the peri-pandemic deployment cycle covering deployment-related quarantining.

### 4.2 Future research

Summing up avenues for future research, we recommend following up on the long-term development of perceived social support for military personnel. The quality of research could be strengthened by comparing pre-pandemic, peri-pandemic, and post-pandemic mental health trajectories. A longer-term post-pandemic follow-up of mental health (Mini-SCL) for soldiers and the civilian population would allow us to assess whether post-pandemic T-values (Mini-SCL) for active military service members return to a more resilient level than the average population, as was the case for pre-pandemic mental health (77). In addition, it is recommended to identify risk and resilience factors that allow the prediction of different resilience trajectories across the deployment cycle. This would provide the opportunity for tailoring prevention strategies.

The generalizability could be tested by evaluating civilian quarantining under ideal quarantining conditions. Research instruments should be harmonized and eventually shortened, facilitating comparisons and reducing missing data and dropouts. In addition, a larger number of research personnel, incentives for continued participation, and over recruitment of young enlisted personnel could contribute as well. These bigger more representative sample sizes would be paramount for predicting resilience trajectories and improved early-on screening for risk allowing for more targeted interventions and respective preventive measures.

## 5 Conclusion

Although these results are indicative that deployment-related quarantining did not have an adverse mental health impact on average, one has to keep in mind the high financial and personnel resources invested to compensate for potential risk factors. The outcome cannot be taken as a given. In addition, this study showed that specific quarantine-related risk and resilience factors are related to mental health in the short and long term, in particular, “clear communication of the quarantine protocol” and “perceived stigma.” The positive message is that one main predictor can be easily addressed: the clear communication of the quarantine protocol, including the purpose and the rules of quarantining. While health-promoting leadership is not the over-arching main predictor or moderator of mental health during the COVID-19 pandemic as in a survey of 7,829 US Army personnel (27), it is also positively related to mental health as it is to the predictive resilience factors of perceived social support, perceived unit cohesion, and clear communication of the quarantine protocol (see Table in Supplementary material 5). While directly influencing the more general resilience factors of perceived social support and perceived unit cohesion might be difficult, health-promoting leadership, involving clearly communicated information and addressing the practicalities of quarantining, is recommended as one important entry point to facilitate the health protective factors of perceived unit cohesion and perceived social support and mental health. As for perceived quarantine-related stigma, this could be expected stigma in high contrast to actual stigma (78) or perceived stigma based on actual experience with fellow soldiers. While it would be useful to differentiate both kinds of stigma, both can be addressed by health-promoting leadership of military leaders as well. Military psychologists could assist in differentiating between actual and expected stigma by fellow soldiers to devise communication strategies for specific target groups, e.g., feedback about non-existent stigma for quarantinees or by also addressing actual stigmatization by fellow soldiers.

## Data availability statement

The datasets presented in this article are not readily available because ownership belongs to the German Ministry of Defense. Requests to access the datasets should be directed to gerddieterwillmund@bundeswehr.org, antjeheikebuehler@bundeswehr.org.

## Ethics statement

The studies involving humans were approved by Charité Ethics Committee, Ethics’ Approval of registered research proposal EA1/388/20. The studies were conducted in accordance with the local legislation and institutional requirements. The participants provided their written informed consent to participate in this study.

## Author contributions

AB: Conceptualization, Data curation, Formal analysis, Funding acquisition, Investigation, Methodology, Project administration, Visualization, Writing – original draft. G-DW: Funding acquisition, Writing – review & editing, Methodology, Resources.
