# Supplementary material for: Deployment-related quarantining—a risk or resilience factor for German military service members? A prospective analysis during the third–fifth waves of COVID-19
Source: Front Public Health. 2023 Dec 13;11:1267581. doi: 10.3389/fpubh.2023.1267581 (PMC10751356; doi:10.3389/fpubh.2023.1267581)
Supplement: Supplementary file 1 [file Data_Sheet_1.pdf]

Supplementary Material 1: Glossary

DEPLOYMENT-RELATED QUARANTINING - A RISK OR RESILIENCE FACTOR?

Antje H. Bühler*, Gerd-Dieter Willmund

*** Correspondence:** anb@ptzbw.org, antjeheikebuehler@bundeswehr.org

1) ISOLATION – QUARANTINE – PRE- AND POST-DEPLOYMENT QUARANTINE:

Isolation is a temporary measure ordered by the civilian authorities in case of a confirmed infection with SARS-CoV-2 [20]. Depending on the severity of the infection, the isolation can be implemented at home or undergoing stationary treatment.

Quarantine is a temporary measure usually ordered by the civilian authorities in case of a confirmed contact with a person who has been infected by the SARS-Cov-2 [20]. In Germany, most often quarantining is implemented at home.

Pre-deployment quarantine during the COVID-pandemic ~~is a measure~~ ordered by the respective Departments or Ministries of Defense following recommendations/directives by the different bodies of the United Nations (UN), including the World Health Organization (WHO) and the UN Department of Peace Operations and Department of Operational Support [16].

It is implemented immediately before deploying to the operational theatre. It is not related to a confirmed infection or a confirmed contact with an infected person. The purpose is to protect vulnerable populations in conflict-ridden countries as well as ensuring operational capabilities of the deploying troops. In Germany, pre-deployment quarantine of soldiers has been implemented as individual confinement to a hotel room.

Post-deployment-quarantine during the COVID-pandemic is a measure ordered by the respective Departments or Ministries of Defense. It is implemented immediately upon returning home, in Germany most often in the form of an at-home- quarantine. Post-deployment quarantine is also not due to a confirmed infection or the confirmed contact with an infected person. Post-deployment quarantine has been suspended before pre-deployment quarantine.

2) RISK AND RESILIENCE FACTORS

Boredom

While in positive psychology, boredom is also described as an opportunity for personal development, and creativity, in clinical psychology and psychiatry, boredom is described as a state of being weary and restless through lack of stimulation and interest, a lack of motivated goal-directed behaviour and sustained attention [15]. It is theoretically and empirically linked to depression through failures of attention and concentration [8] and empirically linked to substance abuse [4] and attention deficit hyperactivity disorder (ADHD) [15]. In the case of quarantining, the lack of external stimulation can result in both. While we conceptualize and operationalize the whole spectrum of boredom, here, it is named as a quarantine-related risk factor based on empirical evidence to date [12, 5].

Health promoting leadership

only refers to military leaders and supervisors as agents in the military context and is more domain-specific, only relating to health promotion. General health promoting leadership aims at raising health awareness, motivation for engaging in healthy behaviours, promoting a health-promoting culture and climate, also leading by example [10, 13, 22]. In the case of the COVID-pandemic, health-promoting leadership behaviours have been conceptualized very specifically in respect to COVID-19-pandemic health behaviours [1]. We conceptualize health promoting leadership by two dimensions, (1) military leaders being perceived to care about the general physical and mental health of their subordinated soldiers and (2) COVID-specific behaviour, including leading by example [7, 6].

Military-specific social support

includes health promoting leadership and perceived unit cohesion.

Need for intimacy and bonding

The „need for intimacy and bonding“ relating to one of the major human needs and motives [11] is conceptualized quarantine-specific as how well quarantine management allows to compensate constraints on fulfilling the need for intimacy and bonding.

Perceived social support

Includes emotional, instrumental support and social connectedness/isolation by others in general.

Perceived stigma

In this study, perceived stigma is defined as perceptions of a person that relevant people see or treat them as someone who has violated the social norms of a relevant group. Therefore, perceived stigma and perceived social norms are interrelated concepts. Both are related to the need of being regarded positively by relevant persons or groups. Based on sociological research, relevant groups or persons for deployed soldiers are the family and fellow soldiers [17]. If social norms are perceived to be

quarantine-conducive, quarantining is expected to be perceived as less stigmatizing. Quarantine-related stigma has been widely-reported, in particular for medical staff [5]. Unlike medical staff, deployed soldiers are not quarantined based on individual infections or contact with an infected person, but due to fulfilling their duty. We do not endorse the view that stigma necessarily is due to the fear of being infected, as stigma has also been encountered being a barrier for seeking help when suffering from mental health conditions [18]. Nonetheless, deployment-related quarantining can result in an extra-burden of workload for fellow soldiers resulting in (perceived) negative views of the quarantinees. Therefore, we conceptualize perceived stigma as the perception that fellow soldiers treat or see them in a more negative way.

Perceived unit cohesion

has been mainly conceptualized and operationalized as perceived social support by military peers and supervisors [14, 9, 21]

Protective factor

A health protective factor is defined as a predictor for physical or mental health. Though defined as a predictor, more often correlations indicate a relationship than a proven causality.

In spite of differing definitions and conceptualizations, we use the terms health protective factors and resilience factors interchangeably. We define risk and protective influence as two ends of a continuum, unless empirically supported otherwise (see risk factor).

Resilience factor

The terms protective factor and resilience factors are used interchangeably by the authors of this article.

Risk factor

Risk factors are defined as predictors for illness, developmental or mental disorders, though more often correlations indicate relationships instead of a proven causality. Most often, they are classified into three groups: a) biological or genetic, b) behavioral or c) environmental risk factors.

Though risk factors constitute an integral element of pathogenetic models and protective factors constitute an integral element of salutogenetic models, we conceptualize risk and protective factors as two ends of a continuum, unless empirically supported otherwise: E. g. social support is seen as a protective factor while lacking social support as a risk factor.

Salutogenesis

In Antonovsky’s concept of salutogenesis [2], mental health does not depend on stressors in a linear way. Mental health or becoming healthy also depends on coping with a state of tension or stress

resulting from the stressors. Coping can be facilitated contextual factors such as e.g. (perceived) social support as well as individual factors.

Sense of coherence

A central concept of Antonovsky's concept of salutogenesis is the individual factor "sense of coherence" [3]. Antonovsky defines sense of coherence as "*a global orientation that expresses the extent to which one has a pervasive, enduring though dynamic feeling of confidence that (1) the stimuli, deriving from one's internal and external environments in the course of living are structured, predictable and explicable* [comprehensibility]*; (2) the resources are available to one to meet the demands posed by these stimuli* [manageable]*; and (3) these demands are challenges, worthy of investment and engagement"* [meaningfulness] [2, 3]. While initially sense of coherence was conceptualized as a stable trait, in particular in adulthood, more recent empirical studies showed that sense of coherence can be affected negatively by traumatic events as well as strengthened by interventions [19].

Literature

1. Adler AB, Gutierrez IA, Gomez SAQ et al.: US soldiers and the role of leadership: COVID-19, mental health, and adherence to public health guidelines. BMC Public Health 2022; 22 (1): 943
2. Antonovsky A: Unravelling the mystery of health: How people manage stress and stay well. San Francisco, CA, US: Jossey-Bass
3. Antonovsky A: The structure and properties of the sense of coherence scale. Social Science and Medicine 1993; 36 (6): 725-733
4. Biolcati R, Mancini G, Trombini E: Proneness to Boredom and Risk Behaviors During Adolescents' Free Time. Psychol Rep 2018; 121 (2): 303-323
5. Brooks SK, Webster RK, Smith LE et al.: The psychological impact of quarantine and how to reduce it: Rapid review of the evidence. The Lancet 2020; 395 (10227): 912-920
6. Bühler A, Willmund G: Adherence and Psychosocial Well-Being During Pandemic-Associated Pre-deployment Quarantine. Front Public Health 2021; 9: 802180
7. Bühler, A., Wesemann, U., Willmund, GD.: Isolierte Unterbringung und häusliche Absonderung: - ein Werkzeugkasten zur Untersuchung von Quarantäneadhärenz, quarantäne-assoziierten und militär-spezifischen Einflussfaktoren. Wehrmedizinische Monatszeitschrift 2022; 66 (2): 62-70
8. Carriere JSA, Cheyne JA, Smilek D: Everyday attention lapses and memory failures: the affective consequences of mindlessness. Conscious Cogn 2008; 17 (3): 835-847
9. Du Preez J, Sundin J, Wessely S, Fear NT: Unit cohesion and mental health in the UK armed forces. Occup Med (Lond) 2012; 62 (1): 47-53
10. Franziska Franke, Jörg Felfe, Alexander Pundt: The impact of health-oriented leadership on follower health:: Development and test of a new instrument measuring health-promoting leadership. Zeitschrift für Personalforschung 2014; 28 (1-2): 139-161
11. Grawe K: Psychologische Therapie. Göttingen: Hogrefe

12. Henssler J, Stock F, van Bohemen J et al.: Mental health effects of infection containment strategies: quarantine and isolation-a systematic review and meta-analysis. Eur Arch Psychiatry Clin Neurosci 2021; 271 (2): 223-234
13. Horstmann D, Remdisch S: Drivers and barriers in the practice of health-specific leadership: A qualitative study in healthcare. Work 2019; 64 (2): 311-321
14. Kanesarajah J, Waller M, Zheng WY, Dobson AJ: Unit cohesion, traumatic exposure and mental health of military personnel. Occup Med (Lond) 2016; 66 (4): 308-315
15. Malkovsky E, Merrifield C, Goldberg Y, Danckert J: Exploring the relationship between boredom and sustained attention. Exp Brain Res 2012; 221 (1): 59-67
16. Nay Minn Tun: Revised Measures for Uniformed Personnel Rotations in a Covid-19 Environment ALL Missions (DPKO-DPA) Circular Code Cable Template
17. Pietsch C.: Zur Motivation deutscher Soldatinnen und Soldaten fürden Afghanistaneinsatz. In: Seiffert A, Langer PC, Pietsch C (Hrsg.): Der Einsatz der Bundeswehr in Afghanistan. Wiesbaden; 101–122
18. Schuy K, Brants LM, Dors S et al.: Mental Health Stigma: An Influencing Factor in Healthcare Utilization by Veterans of the German Armed Forces. Gesundheitswesen 2019; 81 (8-09): e146-e153
19. Super S, Wagemakers, M. A., Koelen, M. A.E., Picavet HSJ, Verkooijen: Strengthening sense of coherence: opportunities for theory building in health promotion. Health Promotion International 2016; 31 (4): 869-878
20. Tipps für die Zeit in häuslicher Quarantäne oder Isolierung: Informationen und Empfehlungen für eine herausfordernde Zeit. Bundeszentrale für gesundheitliche Aufklärung
21. Vogt D, Smith BN, King LA et al.: Deployment risk and resilience inventory-2 (DRRI-2): an updated tool for assessing psychosocial risk and resilience factors among service members and veterans. J Trauma Stress 2013; 26 (6): 710-717
22. Yao L, Li P, Wildy H: Health-Promoting Leadership: Concept, Measurement, and Research Framework. Front. Psychol. 2021; 12. https://doi.org/10.3389/fpsyg.2021.602333
