# Supplementary material for: Deployment-related quarantining—a risk or resilience factor for German military service members? A prospective analysis during the third–fifth waves of COVID-19
Source: Front Public Health. 2023 Dec 13;11:1267581. doi: 10.3389/fpubh.2023.1267581 (PMC10751356; doi:10.3389/fpubh.2023.1267581)
Supplement: Supplementary file 2 [file Data_Sheet_2.PDF]

frontiers

# *Supplementary Material 2*

DEPLOYMENT-RELATED QUARANTINING - A RISK OR RESILIENCE FACTOR?

**Antje H. Bühler*, Gerd-Dieter Willmund**

*** Correspondence:** anb@ptzbw.org, antjeheikebuehler@bundeswehr.org

## Required sample sizes calculated with the help of GPower

We adjusted the alpha error for multiple testing taking into account a previous publication on the course of pre-deployment quarantine [16]. Required sample sizes were calculated with the help of GPower (66).

*Research question 1 and 2:* Adjusting for repeated non-directional testing, the initial α-error of α = .05 has been set at α = .007 for seven one-way repeated measures ANOVAs. A minimum sample size of 94 participants is needed to provide sufficient power (1-β err prob = 0.80) for detecting differences of a medium effect size (f = 0.15) in mental health, perceived social support and perceived unit cohesion across pre- and post-deployment quarantine based on four measurements; a minimum sample size of 113 is needed to detect differences between pre-deployment quarantine and three months post-deployment (effect size $f$ = 0.15, α err prob = 0.007, power (1-β err prob) = 0.80, number of groups = 1, number of measurements = 3, corr among rep measures = 0.5).
As for predicting mental health at the end of post-deployment quarantine and three months post-deployment, the underlying hypotheses on the relationship between mental health and risk and resilience factors are directed, the initial error probability is set at α = 0.1. For predicting mental health at the end of post-deployment quarantine and three months post-deployment, two more linear multiple stepwise regressions are carried out in addition to three previous ones [16]. The error probability is adjusted accordingly at α = 0.02. For detecting a medium effect size ($f^2$ = 0.15), the a priori computed required sample size is 143 and for a large effect size ($f^2$ = 0.35), the a-priori computed sample size is 76 (F-tests—Linear multiple regression: Fixed model, $R^2$ deviation from zero, effect size $f^2$ = 0.15, α err prob = 0.02, power (1-β err prob) = .80, number of predictors = 13). The power is not sufficient for detecting a small effect size ($f^2$ = 0.02), for which the priori computed required sample size is 1092.
