# Supplementary material for: Deployment-related quarantining—a risk or resilience factor for German military service members? A prospective analysis during the third–fifth waves of COVID-19
Source: Front Public Health. 2023 Dec 13;11:1267581. doi: 10.3389/fpubh.2023.1267581 (PMC10751356; doi:10.3389/fpubh.2023.1267581)
Supplement: Supplementary file 3 [file Data_Sheet_3.PDF]

Supplementary Material 3

DEPLOYMENT-RELATED QUARANTINING - A RISK OR RESILIENCE FACTOR?

Antje H. Bühler*, Gerd-Dieter Willmund

*** Correspondence:** anb@ptzbw.org, antjeheikebuehler@bundeswehr.org

**Supplementary Table 1**	Correlations between psychosocial outcome variables and dropout three months post-deployment ($r_{t5)}$ respectively suspended post-deployment quarantine (r_{t3t5})					
	Drop-out at t5			No participation in post-deployment quarantine/study		
T2	r_{t2t5}	*p*	*N*	r_{t3t5}	*p*	*N*
Mini-SCL	-.021	.458	907	.076	.118	303
FSozU-K22	-.016	.564	903	-.010	.830	308
Unit cohesion	.022	.431	904	-.084	.076	303

**Supplementary Table 2**	Correlations between quarantine-associated factors (t2) and dropout three months post-deployment ($r_{t5)}$ respectively suspended post-deployment quarantine (r_{t4})					
	Drop-out at t5			No participation in post-deployment quarantine/study		
T2	r_{t5}	*p*	*N*	r_{t2t3}	*p*	*N*
Informedness COVID	-.037	.174	926	-.028	.572	276
Clear quarantine protocol	-.005	.868	924	-.045	.369	276
Perceived infection risk	-.054*	.048	919	-.045	.366	275
Social norms	-.001	.975	921	-.118*	.017	274
Stigma	.044	.128	920	.062	.233	275
Benefit quarantine	-.021	.434	923	-.087	.080	276
Practicalities	-.039	.150	923	-.005	.916	275
Intimacy/Bonding	-.031	.247	922	-.144**	.004	274
Boredom	-.082	.002	920	-.043	.389	
Financial disadvantage				-.014	.817	274
Health promoting leadership	.024	.393	911	-.059	.247	273

**Supplementary Table 3**	Correlations between socio-demographic variables and dropout three months post-deployment ($r_{t5)}$ respectively suspended post-deployment quarantine (r_{t3t5})					
	Drop-out at t5			Participation in post-deployment quarantine/study		
	r_{t5}	p	N	r_{t4}	p	N
Age	-.139***	< .001	926	-.078**	.004	926
Sex/gender	.016	.635	928	-.007	.838	928
Rank	-.077*	.016	895	-.120***	<.001	895
Partnership	-.031	.341	925	-.022	.501	925
Number of children	-.67*	.030	921	.031	.309	921
Single care taker	.017	.688	590	-.037	.363	590
Children in COVID-related emergency care	-.038	.357	585	.013	.745	585
times deployed	-.082	.005	901	-.044	.133	901
Accumulated days deployed	.076**	.008	881	-.011	.702	881
Times quarantined (t1)	-.073	.062	626	-.056	.152	626
Accumulated days of quarantining (t1)	.018	.594	633	.024	.475	633
Times quarantined (t5)	-	-	-	-.292***	< .001	278
Accumulated days of quarantining (t5)	-	-	-	-.267***	< .001	256
