# Supplementary material for: Deployment-related quarantining—a risk or resilience factor for German military service members? A prospective analysis during the third–fifth waves of COVID-19
Source: Front Public Health. 2023 Dec 13;11:1267581. doi: 10.3389/fpubh.2023.1267581 (PMC10751356; doi:10.3389/fpubh.2023.1267581)
Supplement: Supplementary file 4 [file Data_Sheet_4.PDF]

Supplementary Material 4

DEPLOYMENT-RELATED QUARANTINING - A RISK OR RESILIENCE FACTOR?

Antje H. Bühler*, Gerd-Dieter Willmund

*** Correspondence:** anb@ptzbw.org, antjeheikebuehler@bundeswehr.org

Supplementary table 4: Description of four mental health trajectories (Mini-SCL GSI): General Severity Index

	improvement (*n* = 49)	deterioriation (*n* = 14)	stable chronic (*n* = 38)	Stable resilient (*n* = 201)
**Sex**				
female	3 (6.1Im%)	3 (21.4%)	3 (7.9%)	27 (13.4%)
male	46 (93.9%)	11 (78.6%)	35 (92.1%)	174 (86.6%)
**Partnership**				
Missing	0	0	0	1
no	14 (28.6%)	7 (50.0%)	7 (18.4%)	23 (11.5%)
yes	35 (71.4%)	7 (50.0%)	31 (81.6%)	177 (88.5%)
**Single caretaker**				
Missing	49	13	36	197
yes	0	1 (100.0%)	2 (100.0%)	4 (100.0%)
**Rank**				
Missing	4	0	1	3
enlisted	5 (11.1%)	0 (0.0%)	3 (8.1%)	14 (7.1%)
NCO	20 (44.4%)	9 (64.3%)	20 (54.1%)	105 (53.0%)
officer	20 (44.4%)	5 (35.7%)	14 (37.8%)	79 (39.9%)
**Age**				
Count	49	14	38	201
Mean (SD)	36.898 (8.954)	35.286 (6.810)	37.632 (8.432)	38.294 (8.773)
Median	36.000	32.000	35.500	37.000
Q1,Q3	31.000, 42.000	30.000, 41.250	31.250, 43.500	32.000, 44.000

Supplementary table 4: Description of four mental health trajectories (Mini-SCL GSI): General Severity Index

	improvement (*n* = 49)	deterioriation (*n* = 14)	stable chronic (*n* = 38)	Stable resilient (*n* = 201)
**Number of children**				
Count	49	14	38	199
Missing	0	0	0	2
Mean (SD)	0.898 (1.123)	0.643 (0.842)	0.947 (1.064)	1.085 (1.081)
Median	0.000	0.000	0.500	1.000
Q1,Q3	0.000, 2.000	0.000, 1.000	0.000, 2.000	0.000, 2.000
**Number of deployments at t1**				
Count	49	14	37	195
Missing	0	0	1	6
Mean (SD)	2.571 (3.524)	1.571 (1.869)	3.081 (4.798)	3.431 (4.561)
Median	1.000	1.000	1.000	2.000
Q1,Q3	0.000, 4.000	0.000, 2.750	0.000, 3.000	1.000, 4.000
**Accumulated days of deployments at t1**				
Count	48	14	36	192
Missing	1	0	2	9
Mean (SD)	211.604 (262.661)	217.643 (326.492)	355.806 (441.862)	307.656 (397.451)
Median	127.500	122.500	167.500	191.500
Q1,Q3	0.000, 265.000	0.000, 322.500	0.750, 600.000	68.250, 400.000
**times quarantined at t1**				
Count	31	8	25	132
Missing	18	6	13	69
Mean (SD)	1.194 (0.477)	1.250 (0.707)	1.600 (0.957)	1.455 (0.714)
Median	1.000	1.000	1.000	1.000

Supplementary table 4: Description of four mental health trajectories (Mini-SCL GSI): General Severity Index

	improvement (*n* = 49)	deterioriation (*n* = 14)	stable chronic (*n* = 38)	Stable resilient (*n* = 201)
Q1,Q3	1.000, 1.000	1.000, 1.000	1.000, 2.000	1.000, 2.000
**Accumulated days of quarantining at t1**				
Count	33	8	27	141
Missing	16	6	11	60
Mean (SD)	7.576 (8.471)	8.375 (8.262)	28.259 (60.379)	13.156 (13.937)
Median	1.000	8.500	14.000	12.000
Q1,Q3	0.000, 14.000	0.750, 14.000	1.000, 22.000	1.000, 20.000
**Times quarantined at t5**				
Count	44	11	33	175
Missing	5	3	5	26
Mean (SD)	1.614 (0.784)	1.364 (0.505)	1.879 (1.053)	1.806 (0.987)
Median	1.000	1.000	1.000	2.000
Q1,Q3	1.000, 2.000	1.000, 2.000	1.000, 3.000	1.000, 2.000
**accumulated days of quarantining at t5**				
Count	41	10	28	164
Missing	8	4	10	37
Mean (SD)	20.146 (8.218)	18.400 (6.786)	28.071 (24.510)	26.213 (21.366)
Median	14.000	14.000	21.500	23.500
Q1,Q3	14.000, 28.000	14.000, 24.000	14.000, 32.000	14.000, 28.000
**Perceived social support (FSozU-K22) at t1**				
Count	49	14	37	200
Missing	0	0	1	1
Mean (SD)	0.001 (0.510)	-0.088 (0.445)	-0.450 (0.691)	0.052 (0.450)

Supplementary table 4: Description of four mental health trajectories (Mini-SCL GSI): General Severity Index

	improvement (*n* = 49)	deterioriation (*n* = 14)	stable chronic (*n* = 38)	Stable resilient (*n* = 201)
Median	0.184	0.037	-0.392	0.193
Q1,Q3	-0.078, 0.322	-0.438, 0.311	-0.958, 0.169	-0.080, 0.343
**Perceived social support t2**				
Count	44	12	35	177
Missing	5	2	3	24
Mean (SD)	-0.026 (0.656)	-0.103 (0.477)	-0.462 (0.828)	0.092 (0.459)
Median	0.222	-0.072	-0.248	0.263
Q1,Q3	-0.444, 0.374	-0.404, 0.299	-1.002, 0.115	-0.086, 0.371
**Perceived social support t5**				
Count	49	14	38	199
Missing	0	0	0	2
Mean (SD)	0.020 (0.521)	0.014 (0.448)	-0.574 (0.728)	0.095 (0.483)
Median	0.237	-0.195	-0.457	0.273
Q1,Q3	-0.194, 0.422	-0.291, 0.395	-1.201, -0.011	-0.162, 0.422
**Perceived unit cohesion t1**				
Count	46	13	36	195
Missing	3	1	2	6
Mean (SD)	0.085 (0.792)	-0.209 (0.784)	-0.333 (0.876)	0.002 (0.791)
Median	0.163	-0.019	-0.282	0.094
Q1,Q3	-0.178, 0.628	-0.628, 0.339	-0.961, 0.259	-0.386, 0.667
**Perceived unit cohesion t2**				
Count	44	12	35	177
Missing	5	2	3	24
Mean (SD)	0.023 (0.859)	-0.193 (0.831)	-0.416 (0.794)	0.030 (0.767)

Supplementary table 4: Description of four mental health trajectories (Mini-SCL GSI): General Severity Index

	improvement (*n* = 49)	deterioriation (*n* = 14)	stable chronic (*n* = 38)	Stable resilient (*n* = 201)
Median	0.189	0.002	-0.384	0.115
Q1,Q3	-0.552, 0.748	-0.726, 0.215	-0.914, 0.173	-0.395, 0.665
**Perceived unit cohesion t3**				
Count	17	4	12	65
Missing	32	10	26	136
Mean (SD)	0.197 (0.630)	0.195 (0.574)	-0.475 (1.119)	0.075 (0.708)
Median	0.358	0.078	-0.484	0.045
Q1,Q3	-0.205, 0.726	-0.035, 0.308	-1.304, 0.449	-0.394, 0.726
**Feeling well-informed about Covid at t1**				
Count	49	14	38	200
Missing	0	0	0	1
Mean (SD)	0.194 (0.831)	-0.064 (0.852)	-0.157 (0.791)	0.080 (0.816)
Median	0.330	0.273	-0.129	0.116
Q1,Q3	-0.361, 0.793	-0.205, 0.451	-0.432, 0.395	-0.428, 0.793
**Feeling well-informed about Covid at t2**				
Count	45	12	35	181
Missing	4	2	3	20
Mean (SD)	0.143 (0.780)	0.065 (0.753)	-0.291 (0.733)	0.075 (0.855)
Median	0.304	0.304	-0.299	0.156
Q1,Q3	-0.378, 0.583	-0.325, 0.403	-0.731, 0.330	-0.459, 0.771

Supplementary table 4: Description of four mental health trajectories (Mini-SCL GSI): General Severity Index

	improvement (*n* = 49)	deterioriation (*n* = 14)	stable chronic (*n* = 38)	Stable resilient (*n* = 201)
**Clear communication of the quarantine protocol t1**				
Count	48	14	38	200
Missing	1	0	0	1
Mean (SD)	0.015 (0.508)	0.105 (0.514)	-0.318 (0.720)	0.052 (0.554)
Median	0.063	0.323	-0.187	0.207
Q1,Q3	-0.398, 0.467	-0.314, 0.437	-0.674, 0.145	-0.260, 0.472
**Clear communication of the quarantine protocol t2**				
Count	45	12	35	181
Missing	4	2	3	20
Mean (SD)	0.236 (0.422)	-0.001 (0.571)	-0.494 (0.844)	0.055 (0.523)
Median	0.338	0.052	-0.247	0.155
Q1,Q3	-0.044, 0.546	-0.425, 0.447	-0.829, 0.029	-0.272, 0.468
**Subjective social norms of relevant others at t1**				
Count	49	14	38	198
Missing	0	0	0	3
Mean (SD)	0.070 (0.698)	0.068 (0.581)	-0.306 (0.662)	-0.003 (0.687)
Median	0.126	0.111	-0.233	0.005
Q1,Q3	-0.427, 0.712	-0.269, 0.430	-0.678, 0.064	-0.473, 0.491

Supplementary table 4: Description of four mental health trajectories (Mini-SCL GSI): General Severity Index

	improvement (*n* = 49)	deterioriation (*n* = 14)	stable chronic (*n* = 38)	Stable resilient (*n* = 201)
**Subjective social norms of relevant others at t2**				
Count	44	12	35	180
Missing	5	2	3	21
Mean (SD)	0.117 (0.680)	0.164 (0.477)	-0.238 (0.655)	0.021 (0.715)
Median	0.319	0.208	-0.169	0.017
Q1,Q3	-0.438, 0.672	0.092, 0.430	-0.643, 0.189	-0.482, 0.598
**Perceived stigmatization by fellow soldiers at t1**				
Count	48	14	37	195
Missing	1	0	1	6
Mean (SD)	-0.066 (0.969)	-0.262 (0.754)	-0.345 (0.762)	0.110 (0.530)
Median	0.118	0.000	0.000	0.235
Q1,Q3	0.000, 0.471	-0.499, 0.294	-0.936, 0.149	0.000, 0.471
**Perceived stigmatization by fellow soldiers at t2**				
Count	45	12	35	180
Missing	4	2	3	21
Mean (SD)	-0.099 (0.895)	-0.291 (0.763)	-0.439 (0.857)	0.054 (0.655)
Median	0.134	-0.010	-0.277	0.256
Q1,Q3	0.012, 0.501	-0.850, 0.317	-0.805, 0.134	0.005, 0.501

Supplementary table 4: Description of four mental health trajectories (Mini-SCL GSI): General Severity Index

	improvement (*n* = 49)	deterioriation (*n* = 14)	stable chronic (*n* = 38)	Stable resilient (*n* = 201)
**Perceived risk of infection at t1**				
Count	49	14	37	200
Missing	0	0	1	1
Mean (SD)	-0.081 (0.821)	0.081 (0.655)	0.261 (0.679)	0.033 (0.673)
Median	-0.105	0.125	0.196	0.018
Q1,Q3	-0.481, 0.377	-0.387, 0.738	-0.283, 0.883	-0.352, 0.533
**Perceived risk of infection at t2**				
Count	45	12	35	180
Missing	4	2	3	21
Mean (SD)	-0.041 (0.694)	0.075 (0.573)	0.305 (0.714)	0.060 (0.679)
Median	-0.138	-0.030	0.349	0.032
Q1,Q3	-0.353, 0.366	-0.302, 0.579	-0.103, 0.739	-0.339, 0.548
**Perceived benefit/effectiveness of quarantining at t1**				
Count	49	14	37	200
Missing	0	0	1	1
Mean (SD)	-0.000 (0.838)	0.082 (0.675)	-0.249 (0.823)	0.042 (0.739)
Median	0.355	-0.034	-0.133	0.145
Q1,Q3	-0.616, 0.655	-0.230, 0.746	-0.644, 0.236	-0.460, 0.610

Supplementary table 4: Description of four mental health trajectories (Mini-SCL GSI): General Severity Index

	improvement (*n* = 49)	deterioriation (*n* = 14)	stable chronic (*n* = 38)	Stable resilient (*n* = 201)
**Perceived benefit/effectiveness of quarantining at t2**				
Count	45	12	35	181
Missing	4	2	3	20
Mean (SD)	0.129 (0.735)	0.053 (0.654)	-0.238 (0.802)	0.058 (0.773)
Median	0.360	0.215	-0.338	0.147
Q1,Q3	-0.391, 0.763	-0.273, 0.537	-0.694, 0.433	-0.482, 0.728
**Perceived practicality of quarantining at t1**				
Count	48	14	37	195
Missing	1	0	1	6
Mean (SD)	0.044 (0.790)	0.245 (0.587)	-0.398 (0.836)	0.080 (0.685)
Median	0.193	0.328	-0.301	0.220
Q1,Q3	-0.389, 0.644	-0.221, 0.821	-0.996, 0.099	-0.472, 0.810
**Perceived practicality of quarantining at t2**				
Count	45	12	35	180
Missing	4	2	3	21
Mean (SD)	0.288 (0.518)	0.021 (0.512)	-0.553 (0.843)	0.109 (0.693)
Median	0.452	-0.140	-0.411	0.318
Q1,Q3	0.071, 0.666	-0.333, 0.488	-1.026, -0.030	-0.412, 0.824

Supplementary table 4: Description of four mental health trajectories (Mini-SCL GSI): General Severity Index

	improvement (*n* = 49)	deterioriation (*n* = 14)	stable chronic (*n* = 38)	Stable resilient (*n* = 201)
**Fulfilled need for intimacy/bonding at t1**				
Count	49	14	37	198
Missing	0	0	1	3
Mean (SD)	-0.013 (0.696)	0.233 (0.560)	-0.399 (0.570)	0.104 (0.694)
Median	-0.051	0.161	-0.443	-0.033
Q1,Q3	-0.567, 0.554	-0.199, 0.637	-0.737, 0.071	-0.333, 0.571
**Fulfilled need for intimacy/bonding at t2**				
Count	45	12	35	179
Missing	4	2	3	22
Mean (SD)	0.070 (0.778)	0.110 (0.483)	-0.427 (0.470)	0.111 (0.681)
Median	-0.009	0.101	-0.452	0.050
Q1,Q3	-0.593, 0.624	-0.171, 0.450	-0.676, -0.074	-0.437, 0.624
**Financial diasadvantages caused by quarantining at t1**				
Count	49	14	37	199
Missing	0	0	1	2
Mean (SD)	0.008 (1.075)	0.307 (0.290)	-0.291 (1.236)	0.024 (0.958)
Median	0.384	0.384	0.384	0.384
Q1,Q3	0.384, 0.384	0.384, 0.384	-0.701, 0.384	0.384, 0.384

Supplementary table 4: Description of four mental health trajectories (Mini-SCL GSI): General Severity Index

	improvement (*n* = 49)	deterioriation (*n* = 14)	stable chronic (*n* = 38)	Stable resilient (*n* = 201)
**Financial diasadvantages caused by quarantining at t2**				
Count	45	12	35	179
Missing	4	2	3	22
Mean (SD)	0.200 (0.804)	0.386 (0.000)	-0.570 (1.556)	-0.008 (1.004)
Median	0.386	0.386	0.386	0.386
Q1,Q3	0.386, 0.386	0.386, 0.386	-1.407, 0.386	0.386, 0.386
**Quarantine-related boredom at t1**				
Count	49	14	37	199
Missing	0	0	1	2
Mean (SD)	0.097 (0.804)	0.215 (0.806)	-0.225 (0.839)	0.102 (0.855)
Median	0.194	0.282	-0.272	0.204
Q1,Q3	-0.434, 0.689	-0.233, 0.810	-0.754, 0.217	-0.428, 0.696
**Quarantine-related boredom at t2**				
Count	45	12	35	180
Missing	4	2	3	21
Mean (SD)	0.268 (0.757)	0.332 (0.652)	-0.431 (0.874)	0.166 (0.855)
Median	0.399	0.460	-0.569	0.244
Q1,Q3	-0.414, 0.863	0.054, 0.863	-1.121, 0.069	-0.408, 0.863

Supplementary table 4: Description of four mental health trajectories (Mini-SCL GSI): General Severity Index

	improvement (*n* = 49)	deterioriation (*n* = 14)	stable chronic (*n* = 38)	Stable resilient (*n* = 201)
**Health promoting leadership at t1**				
Count	48	14	36	197
Missing	1	0	2	4
Mean (SD)	0.227 (0.632)	-0.513 (1.079)	-0.250 (0.987)	0.054 (0.797)
Median	0.258	-0.410	-0.109	0.040
Q1,Q3	-0.137, 0.842	-0.875, 0.329	-1.063, 0.562	-0.463, 0.840
**Health promoting leadership at t2**				
Count	45	12	35	178
Missing	4	2	3	23
Mean (SD)	0.080 (0.798)	-0.217 (0.880)	-0.649 (0.997)	0.022 (0.844)
Median	0.230	-0.251	-0.738	0.084
Q1,Q3	-0.517, 0.745	-0.956, 0.370	-1.369, 0.240	-0.515, 0.750

1
