# Supplementary material for: Deployment-related quarantining—a risk or resilience factor for German military service members? A prospective analysis during the third–fifth waves of COVID-19
Source: Front Public Health. 2023 Dec 13;11:1267581. doi: 10.3389/fpubh.2023.1267581 (PMC10751356; doi:10.3389/fpubh.2023.1267581)
Supplement: Supplementary file 5 [file Data_Sheet_5.PDF]

Supplementary Material 5

DEPLOYMENT-RELATED QUARANTINING - A RISK OR RESILIENCE FACTOR?

Antje H. Bühler*, Gerd-Dieter Willmund

*** Correspondence:** anb@ptzbw.org, antjeheikebuehler@bundeswehr.org

Supplementary table 5: Correlations of Health promoting leadership with mental health three months post-deployment (t5) and with risk and resilience factors at the end of pre-deployment quarantine (t2)

Correlations

		Zscore: T-value t5 Mini_SCL	zt2_FSozU	zt2_Unit_Cohesion	zt2_Info_Covid	zt2_clear_quarantine_protocol	zt2_social_norms
Pearson Correlation	Zscore: T-value t5 Mini_SCL	1.000	-.270	-.337	-.223	-.344	-.218
	zt2_FSozU	-.270	1.000	.281	.205	.209	.132
	zt2_Unit_Cohesion	-.337	.281	1.000	.274	.284	.192
	zt2_Info_Covid	-.223	.205	.274	1.000	.435	.249
	zt2_clear_quarantine_protocol	-.344	.209	.284	.435	1.000	.395
	zt2_social_norms	-.218	.132	.192	.249	.395	1.000
	zt2_stigma	-.218	.134	.217	.190	.252	.242
	zt2_infection_risk	.146	-.026	-.091	.104	.018	.132
	zt2_benefit_effectiveness	-.143	.112	.162	.364	.395	.554
	zt2_practicality	-.342	.238	.320	.337	.524	.464
	zt2_intimacy_bonding	-.315	.010	.079	.300	.326	.383
	Zscore(t2_FinDisadvantage) EF50	-.184	.073	.121	.107	.130	.213
	zt2_boredom	-.211	.056	.124	.205	.288	.447
	zt2_HealthPromLeader	-.255	.208	.603	.350	.378	.265

Sig. (1-tailed)	Zscore: T-value t5 Mini_SCL	.	<.001	<.001	<.001	<.001	<.001
	zt2_FSozU	.000	.	.000	.000	.000	.016
	zt2_Unit_Cohesion	.000	.000	.	.000	.000	.001
	zt2_Info_Covid	.000	.000	.000	.	.000	.000
	zt2_clear_quarantine_protocol	.000	.000	.000	.000	.	.000
	zt2_social_norms	.000	.016	.001	.000	.000	.
	zt2_stigma	.000	.014	.000	.001	.000	.000
	zt2_infection_risk	.008	.336	.069	.046	.388	.016
	zt2_benefit_effectiveness	.010	.034	.004	.000	.000	.000
	zt2_practicality	.000	.000	.000	.000	.000	.000
	zt2_intimacy_bonding	.000	.434	.101	.000	.000	.000
	Zscore(t2_FinDisadvantage) EF50	.001	.119	.024	.041	.017	.000
	zt2_boredom	.000	.179	.022	.000	.000	.000
	zt2_HealthPromLeader	.000	.000	.000	.000	.000	.000
N	Zscore: T-value t5 Mini_SCL	266	266	266	266	266	266
	zt2_FSozU	266	266	266	266	266	266
	zt2_Unit_Cohesion	266	266	266	266	266	266
	zt2_Info_Covid	266	266	266	266	266	266
	zt2_clear_quarantine_protocol	266	266	266	266	266	266
	zt2_social_norms	266	266	266	266	266	266
	zt2_stigma	266	266	266	266	266	266
	zt2_infection_risk	266	266	266	266	266	266
	zt2_benefit_effectiveness	266	266	266	266	266	266
	zt2_practicality	266	266	266	266	266	266
	zt2_intimacy_bonding	266	266	266	266	266	266
	Zscore(t2_FinDisadvantage)EF50	266	266	266	266	266	266
	zt2_boredom	266	266	266	266	266	266
	zt2_HealthPromLeader	266	266	266	266	266	266

Correlations

		zt2_stigma	zt2_infection_risk	zt2_benefit_effectiveness	zt2_practicality	zt2_intimacy_bonding	Zscore(t2_FinDisadvantage) EF50
Pearson Correlation	Zscore: T-value t5 Mini_SCL	-.218	.146	-.143	-.342	-.315	-.184
	zt2_FSozU	.134	-.026	.112	.238	.010	.073
	zt2_Unit_Cohesion	.217	-.091	.162	.320	.079	.121
	zt2_Info_Covid	.190	.104	.364	.337	.300	.107
	zt2_clear_quarantine_protocol	.252	.018	.395	.524	.326	.130
	zt2_social_norms	.242	.132	.554	.464	.383	.213
	zt2_stigma	1.000	-.019	.142	.263	.311	.312
	zt2_infection_risk	-.019	1.000	.245	-.006	.000	-.079
	zt2_benefit_effectiveness	.142	.245	1.000	.384	.242	.063
	zt2_practicality	.263	-.006	.384	1.000	.288	.176
	zt2_intimacy_bonding	.311	.000	.242	.288	1.000	.254
	Zscore(t2_FinDisadvantage) EF50	.312	-.079	.063	.176	.254	1.000
	zt2_boredom	.219	.030	.345	.445	.396	.130
	zt2_HealthPromLeader	.171	-.014	.326	.428	.166	.141
Sig. (1-tailed)	Zscore: T-value t5 Mini_SCL	<.001	.008	.010	<.001	<.001	.001
	zt2_FSozU	.014	.336	.034	.000	.434	.119
	zt2_Unit_Cohesion	.000	.069	.004	.000	.101	.024
	zt2_Info_Covid	.001	.046	.000	.000	.000	.041
	zt2_clear_quarantine_protocol	.000	.388	.000	.000	.000	.017
	zt2_social_norms	.000	.016	.000	.000	.000	.000
	zt2_stigma	.	.381	.010	.000	.000	.000
	zt2_infection_risk	.381	.	.000	.462	.500	.099
	zt2_benefit_effectiveness	.010	.000	.	.000	.000	.155

	zt2_practicality	.000	.462	.000	.	.000	.002
	zt2_intimacy_bonding	.000	.500	.000	.000	.	.000
	Zscore(t2_FinDisadvantage) EF50	.000	.099	.155	.002	.000	.
	zt2_boredom	.000	.315	.000	.000	.000	.017
	zt2_HealthPromLeader	.003	.411	.000	.000	.003	.011
N	Zscore: T-value t5 Mini_SCL	266	266	266	266	266	266
	zt2_FSozU	266	266	266	266	266	266
	zt2_Unit_Cohesion	266	266	266	266	266	266
	zt2_Info_Covid	266	266	266	266	266	266
	zt2_clear_quarantine_protocol	266	266	266	266	266	266
	zt2_social_norms	266	266	266	266	266	266
	zt2_stigma	266	266	266	266	266	266
	zt2_infection_risk	266	266	266	266	266	266
	zt2_benefit_effectiveness	266	266	266	266	266	266
	zt2_practicality	266	266	266	266	266	266
	zt2_intimacy_bonding	266	266	266	266	266	266
	Zscore(t2_FinDisadvantage) EF50	266	266	266	266	266	266
	zt2_boredom	266	266	266	266	266	266
	zt2_HealthPromLeader	266	266	266	266	266	266

Correlations

		zt2_boredom	zt2_HealthPromLeader
Pearson Correlation	Zscore: T-value t5 Mini_SCL	-.211	-.255
	zt2_FSozU	.056	.208
	zt2_Unit_Cohesion	.124	.603
	zt2_Info_Covid	.205	.350
	zt2_clear_quarantine_protocol	.288	.378
	zt2_social_norms	.447	.265
	zt2_stigma	.219	.171
	zt2_infection_risk	.030	-.014
	zt2_benefit_effectiveness	.345	.326
	zt2_practicality	.445	.428
	zt2_intimacy_bonding	.396	.166
	Zscore(t2_FinDisadvantage) EF50	.130	.141
	zt2_boredom	1.000	.237
	zt2_HealthPromLeader	.237	1.000
Sig. (1-tailed)	Zscore: T-value t5 Mini_SCL	<.001	<.001
	zt2_FSozU	.179	.000
	zt2_Unit_Cohesion	.022	.000
	zt2_Info_Covid	.000	.000
	zt2_clear_quarantine_protocol	.000	.000
	zt2_social_norms	.000	.000
	zt2_stigma	.000	.003
	zt2_infection_risk	.315	.411
	zt2_benefit_effectiveness	.000	.000
	zt2_practicality	.000	.000
	zt2_intimacy_bonding	.000	.003
	Zscore(t2_FinDisadvantage) EF50	.017	.011
	zt2_boredom	.	.000

	zt2_HealthPromLeader	.000	.
N	Zscore: T-value t5 Mini_SCL	266	266
	zt2_FSozU	266	266
	zt2_Unit_Cohesion	266	266
	zt2_Info_Covid	266	266
	zt2_clear_quarantine_protocol	266	266
	zt2_social_norms	266	266
	zt2_stigma	266	266
	zt2_infection_risk	266	266
	zt2_benefit_effectiveness	266	266
	zt2_practicality	266	266
	zt2_intimacy_bonding	266	266
	Zscore(t2_FinDisadvantage) EF50	266	266
	zt2_boredom	266	266
	zt2_HealthPromLeader	266	266

Legend:

z: all values were z-standardized

Zscore: T-value t5 Mini_SCL: Mental health based on sex- and age-specific Tvalues - three months post-deployment (approximately 7 months after pre-deployment quarantine

Social protective factors (general and military-specific): t2

zt2_FSozU: Perceived social support (FSozU-K22)

zt2_Unit_Cohesion: Perceived unit cohesion

Quarantine-specific risk and resilience factors (z-standardized): t2 (end of pre-deployment quarantine)

zt2_Info_Covid: Feeling well-informed about Covid:

zt2_clear_quarantine_protocol: Clear communication of the quarantine protocol

zt2_social_norms: Subjective social norms of relevant other (fellow soldiers, family):

zt2_stigma: Perceived stigmatization by fellow soldiers

zt2_infection_risk: Perceived risk of infection

zt2_benefit_effectiveness: Perceived benefit/effectiveness of quarantining

zt2_practicality: Perceived practicality of quarantining

zt2_intimacy_bonding: Fulfilled need for intimacy/bonding

Zscore(t2_FinDisadvantage) EF50: Financial diasadvantages caused by quarantining

zt2_boredom: Quarantine-related boredom

zt2_HealthPromLeader: Health promoting leadership
