# Supplementary material for: Deployment-related quarantining—a risk or resilience factor for German military service members? A prospective analysis during the third–fifth waves of COVID-19
Source: Front Public Health. 2023 Dec 13;11:1267581. doi: 10.3389/fpubh.2023.1267581 (PMC10751356; doi:10.3389/fpubh.2023.1267581)
Supplement: Supplementary file 6 [file Data_Sheet_6.PDF]

Supplementary Material 6

DEPLOYMENT-RELATED QUARANTINING - A RISK OR RESILIENCE FACTOR?

Antje H. Bühler*, Gerd-Dieter Willmund

*** Correspondence:** anb@ptzbw.org, antjeheikebuehler@bundeswehr.org

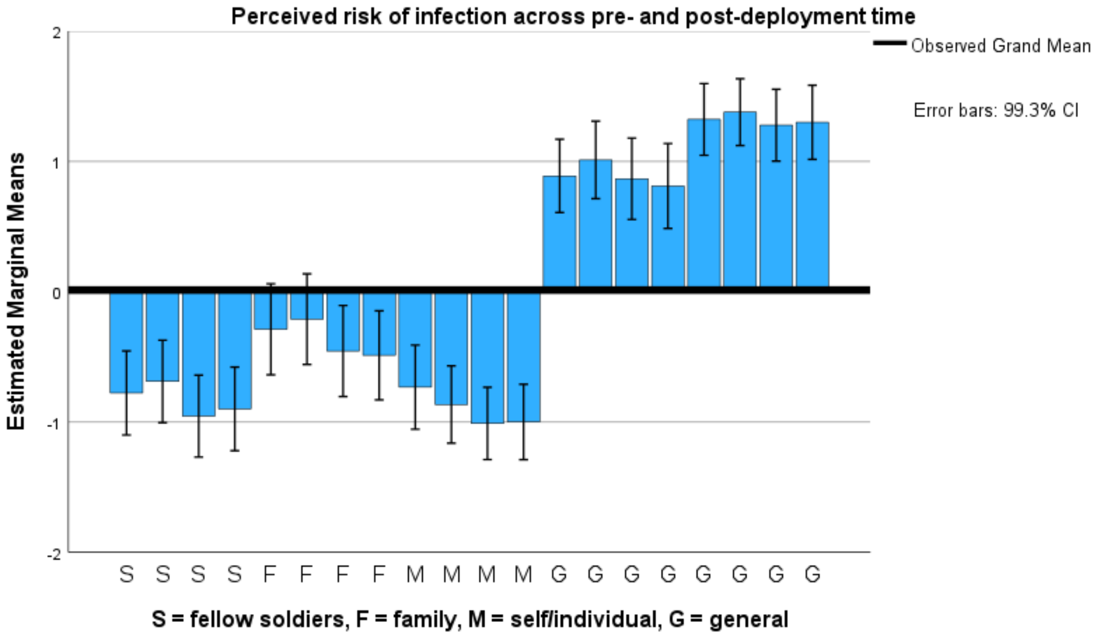

Supplementary Figure 1. Perceived risk of infection for fellow soldiers, family, oneself and general.

Note: Perceived risk of infection for fellow soldiers (S), family (F), self/individual (M) and general perceived risk of infection (G) are assessed for the beginning and end of pre- and post-deployment quarantine, values are displayed in chronological order for each reference group/person
