# Supplementary material for: Deployment-related quarantining—a risk or resilience factor for German military service members? A prospective analysis during the third–fifth waves of COVID-19
Source: Front Public Health. 2023 Dec 13;11:1267581. doi: 10.3389/fpubh.2023.1267581 (PMC10751356; doi:10.3389/fpubh.2023.1267581)
Supplement: Supplementary file 7 [file Data_Sheet_7.PDF]

Supplementary Material 7

DEPLOYMENT-RELATED QUARANTINING - A RISK OR RESILIENCE FACTOR?

Antje H. Bühler*, Gerd-Dieter Willmund

*** Correspondence:** anb@ptzbw.org, antjeheikebuehler@bundeswehr.org

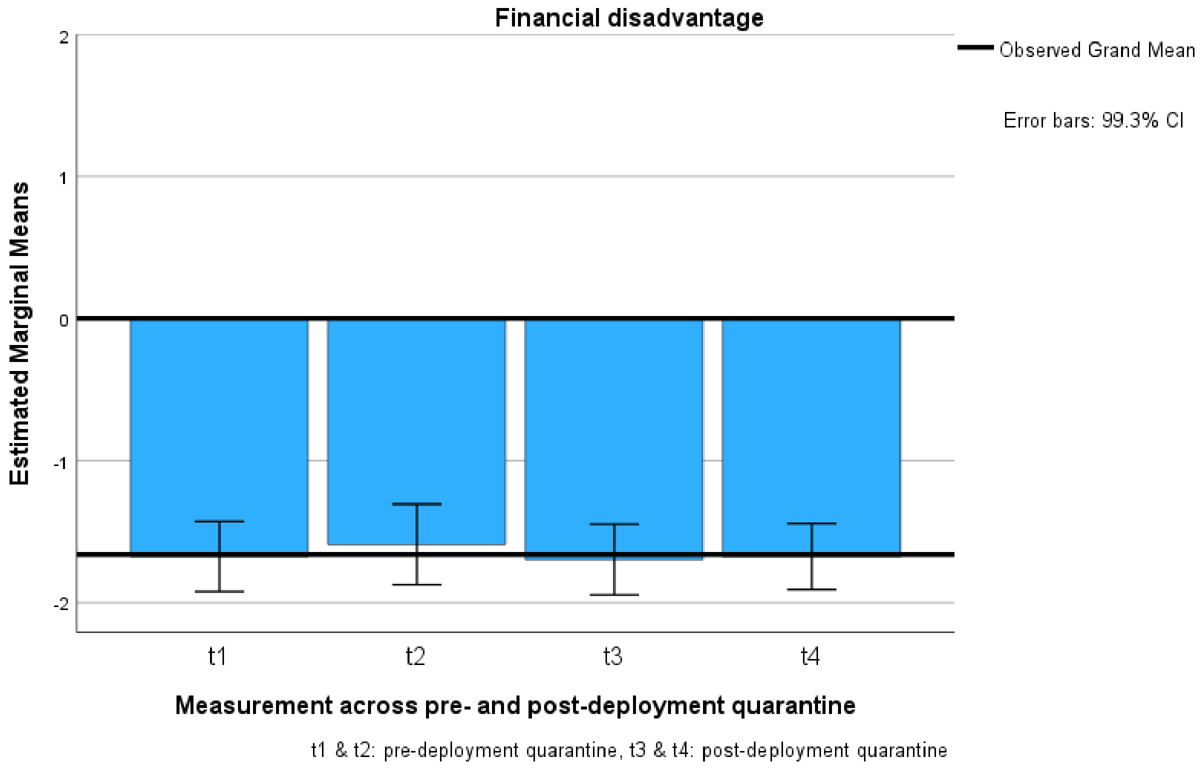

Supplementary Figure 2. Perceived financial disadvantage due to quarantining assessed for the beginning and end of pre- and post-deployment quarantine

Note. The five-point likert-scale ranges between a minimum value of -2 and a maximum value of 2, -2 = no financial advantage at all, +2= high financial disadvantage.
